# Impact of Human FcγR Gene Polymorphisms on IgG-Triggered Cytokine Release: Critical Importance of Cell Assay Format

**DOI:** 10.3389/fimmu.2019.00390

**Published:** 2019-03-07

**Authors:** Khiyam Hussain, Chantal E. Hargreaves, Tania F. Rowley, Joshua M. Sopp, Kate V. Latham, Pallavi Bhatta, John Sherington, Rona M. Cutler, David P. Humphreys, Martin J. Glennie, Jonathan C. Strefford, Mark S. Cragg

**Affiliations:** ^1^Antibody and Vaccine Group, Centre for Cancer Immunology, Cancer Sciences, Faculty of Medicine, University of Southampton, Southampton, United Kingdom; ^2^Nuffield Department of Medicine, John Radcliffe Hospital, University of Oxford, Oxford, United Kingdom; ^3^Cancer Genomics Group, Southampton Experimental Cancer Medicine Centre, Cancer Sciences Unit, Faculty of Medicine, University of Southampton, Southampton, United Kingdom; ^4^UCB Pharma, Slough, United Kingdom

**Keywords:** Fc gamma receptors, antibody immunotherapy, Fc gamma receptor polymorphism, cytokine release syndrome, cytokine release assays

## Abstract

Monoclonal antibody (mAb) immunotherapy has transformed the treatment of allergy, autoimmunity, and cancer. The interaction of mAb with Fc gamma receptors (FcγR) is often critical for efficacy. The genes encoding the low-affinity FcγR have single nucleotide polymorphisms (SNPs) and copy number variation that can impact IgG Fc:FcγR interactions. Leukocyte-based *in vitro* assays remain one of the industry standards for determining mAb efficacy and predicting adverse responses in patients. Here we addressed the impact of FcγR genetics on immune cell responses in these assays and investigated the importance of assay format. FcγR genotyping of 271 healthy donors was performed using a Multiplex Ligation-Dependent Probe Amplification assay. Freeze-thawed/pre-cultured peripheral blood mononuclear cells (PBMCs) and whole blood samples from donors were stimulated with reagents spanning different mAb functional classes to evaluate the association of FcγR genotypes with T-cell proliferation and cytokine release. Using freeze-thawed/pre-cultured PBMCs, agonistic T-cell-targeting mAb induced T-cell proliferation and the highest levels of cytokine release, with lower but measurable responses from mAb which directly require FcγR-mediated cellular effects for function. Effects were consistent for individual donors over time, however, no significant associations with FcγR genotypes were observed using this assay format. In contrast, significantly elevated IFN-γ release was associated with the *FCGR2A*-131H/H genotype compared to *FCGR2A*-131R/R in whole blood stimulated with Campath (*p* ≤ 0.01) and IgG1 Fc hexamer (*p* ≤ 0.05). Donors homozygous for both the high affinity *FCGR2A*-131H and *FCGR3A*-158V alleles mounted stronger IFN-γ responses to Campath (*p* ≤ 0.05) and IgG1 Fc Hexamer (*p* ≤ 0.05) compared to donors homozygous for the low affinity alleles. Analysis revealed significant reductions in the proportion of CD14^hi^ monocytes, CD56^dim^ NK cells (*p* ≤ 0.05) and FcγRIIIa expression (*p* ≤ 0.05), in donor-matched freeze-thawed PBMC compared to whole blood samples, likely explaining the difference in association between FcγR genotype and mAb-mediated cytokine release in the different assay formats. These findings highlight the significant impact of *FCGR2A* and *FCGR3A* SNPs on mAb function and the importance of using fresh whole blood assays when evaluating their association with mAb-mediated cytokine release *in vitro*. This knowledge can better inform on the utility of *in vitro* assays for the prediction of mAb therapy outcome in patients.

## Introduction

The advent of monoclonal antibodies (mAb) has revolutionized the treatment of malignant and autoimmune disease (1, 2). However, there is considerable variability in response to mAb therapy, as some patients may not respond to treatment whilst others experience toxic side effects, of which cytokine release syndrome (CRS) is the most detrimental to patient safety (3). CRS is characterized by rapid immune cell activation and systemic elevations of proinflammatory cytokines, in particular IFN-γ, TNF-α, and IL-6. CRS has been observed with the clinical use of several antibodies, including muromonab (anti-CD3), TGN1412 (anti-CD28), Rituximab (anti-CD20) and alemtuzumab (Campath-1H, anti-CD52) (3–6).

In the first-in-man trial of TGN1412, rapid, life-threatening CRS was observed in healthy volunteers (6). Preclinical *in vitro* testing using soluble TGN1412 to stimulate human whole blood or purified peripheral blood mononuclear cells (PBMCs) failed to predict this toxicity (7). Following these failures, there has been a concerted effort to develop predictive *in vitro* assays that enable a better understanding of mAb *in vivo* action and potential toxicity (8–10). mAb target density, immunoglobulin G (IgG) isotype, tissue microenvironment and Fc gamma receptor (FcγR) expression levels are all key to the outcome of therapy (11). Importantly, several studies have reported that *in vivo* expression levels and distribution of FcγR profoundly influence mAb effector function (12–15). Recapitulating the *in vivo* interaction of the mAb with FcγR *in vitro* is therefore of significant value.

Six FcγR are present in humans, consisting of high and low affinity receptors. The high-affinity FcγRI (CD64) is encoded by *FCGR1A* on chromosome 1q21. The low-affinity receptors, FcγRIIa, FcγRIIb, FcγRIIc, FcγRIIIa, and FcγRIIIb are encoded by genes *FCGR2A, FCGR2B, FCGR2C, FCGR3A*, and *FCGR3B*, respectively, in a 200 kb region on chromosome 1q23-24. These genes are subject to numerous single nucleotide polymorphisms (SNPs) and copy number variation (CNV) (16). There are four reported copy number regions (CNRs) in the low-affinity locus, each encompassing a differing combination of genes (17). Genetic variation can impact upon receptor function and associations have been made between FcγR genetic variants and disease. SNPs in *FCGR2A* (rs1801274; 131H) and *FCGR3A* (rs396991; 158V) increase receptor affinity for IgG (18) while CNV can alter the level of FcγR expressed at the cell surface available for IgG binding. SNPs altering receptor affinity have been associated with superior responses in some cohorts of cancer patients treated with mAb immunotherapy (19–24). *FCGR2A*-131R (25) and *FCGR2B*-232T (26) have been implicated in increased risk of systemic lupus erythematous, while *FCGR3B* HNA 1B and decreased copy number of *FCGR3B* have been associated with reduced immune complex clearance and increased risk of autoimmunity (25, 27).

Given the impact of FcγR SNPs and CNV on receptor function via IgG Fc:FcγR interactions on immune cells (28, 29), studies investigating treatment efficacy and side effect profile in the context of FcγR genotypes and expression levels are warranted. We hypothesized that since the *FCGR2A*-131H and *FCGR3A*-158V alleles in particular, markedly enhance receptor affinity for IgG (18), mAbs and an IgG1 Fc Hexamer construct, have the potential to elicit enhanced cytokine release amongst individuals possessing these gene variants. Furthermore, we aimed to determine the association of several FcγR SNPs with the magnitude of IgG Fc triggered cytokine release in two widely used assay formats: freeze-thawed precultured PBMCs and whole blood. The whole blood assay (but not freeze-thawed PBMC) format recapitulates immune cell subset frequencies, FcγR cellular distribution and expression levels at physiological levels more accurately, revealing associations between FcγR genotype and magnitude of cytokine release in response to mAb treatment. These findings highlight FcγR genotype characterization paired with *vitro* assessment of mAb therapeutics may indeed better predict the magnitude, and variability of responses observed in clinical settings and inform on enhanced therapy design.

## Materials and Methods

### Healthy Donor Cohorts and Ethical Approval

This study comprises two independent cohorts of anonymous healthy donors (total *n* = 271). The Southampton cohort (30), consisted of 178 anonymous healthy donors entering local transfusion services (National Blood Service, Southampton, UK). This study was approved by the University of Southampton Faculty of Medicine Ethics Committee and the National Research Ethics Service Committee South Central, Hampshire, UK. The UCB cohort consisted of 93 anonymous healthy donors based at UCB Celltech, Slough, UK. Blood samples obtained from these donors were taken with informed consent under UCB Celltech UK HTA license number 12504. All donors gave written informed consent in accordance with the Declaration of Helsinki.

### PBMC Preparation and Blood Collection

PBMCs were sourced from leukocyte cones (National Blood Service, Southampton, UK) and whole blood was collected from the UCB donor cohort in lithium heparin vacutainers (BD). PBMCs were isolated from these samples immediately by density gradient centrifugation (Lymphoprep, Axis-Shield). Samples were subsequently frozen in 10% DMSO and 90% fetal bovine serum (FBS, Sigma-Aldrich) and stored in liquid nitrogen for 3–24 months.

### Genomic DNA Extraction and Multiplex Ligation-Dependent Probe Amplification (MLPA) Assay

Frozen PBMC samples were rapidly thawed and genomic DNA (gDNA) was extracted (DNeasy Blood and Tissue Kit, Qiagen, GmbH, Hilden, Germany). DNA quality was assessed by UV spectrophotometry.

CNV and SNPs in the low-affinity FcγR locus were measured as previously described (30). 100 ng DNA was analyzed in triplicate using the SALSA MLPA P110 and P111 probe mixes (MRC-Holland, Amsterdam, The Netherlands). PCR products were analyzed using the Genetic Analysis System CEQ 8800 capillary electrophoresis machine and GenomeLab software (Beckman Coulter, High Wycombe, UK). CNV across the locus and SNPs in *FCGR2A* 131R/H (rs1801274), *FCGR3A* 158F/V (rs396991), *FCGR2B* 232I/T (rs1050501), *FCGR2C* 57X/Q (rs759550223), *FCGR3B* HNA 1A/B/C isoforms were assessed.

Intra-sample data normalization was performed using the Coffalyser.NET software (MRC-Holland) by comparing the peak heights of PCR products generated by probes detecting regions of interest against the peak heights of PCR products targeting control genes of known normal copy number. Inter-sample normalization was performed by comparing test cases against a reference sample of 96 pooled European Collection of Cell Cultures (ECACC) Human Random Control panel 1 (Porton Down, Public Health England, UK) gDNA samples. Normalized MLPA data was analyzed using Microsoft Excel 2010.

### Antibodies and IgG1 Fc Hexamer

Avastin (Bevacizumab) was sourced from Genentech. Hybridoma cells expressing OKT3 (mouse IgG2a) were obtained from the American Type Culture Committee (ATCC) and mAb isolated from tissue culture media by standard procedures in-house. TGN1412 was produced in-house using published sequences (US patent number US7585960). Variable regions were sub-cloned into expression vectors (pEE6.4 heavy chain and pEE12.4 light chain; Lonza) containing constant regions of human IgG4. Heavy- and light-chain vectors were sub-cloned together before transfection into 293F cells for transient production or CHO-K1 cells for stable production. mAb was purified on Protein A-Sepharose, and aggregates were removed by gel filtration. Campath-1H (Campath) human IgG1 was sourced from Professor Geoffrey Hale (University of Cambridge, UK).

To generate a recombinant hexameric Fc construct (IgG1 Fc hexamer), human IgG1 Fc with mature N-termini starting with an IgG1 core hinge (CPPC) were directly fused at their C-terminal lysine residues to the 18 amino-acid C-terminal extension or “tail-piece” (PTLYNVSLVMSDTAGTCY) of human IgM, which promotes covalent multimerization. IgG1 Fc hexamer was expressed transiently in CHO cells and purified using Protein A and S200 size exclusion chromatography as described previously (28, 29). IgG1 Fc hexamer fraction purity was >98% on analytical HPLC after size exclusion chromatography (SEC). IgG1 Fc hexamer was stored at 4°C in PBS or frozen in aliquots at −80°C.

Endotoxin levels for all antibodies and IgG1 Fc Hexamer used in this study were measured and found to be <1 ng/mg protein (Endosafe-PTS, Charles River Laboratories).

### PBMC Assay

Frozen PBMCs were rapidly thawed at 37°C and cultured for 24 h in a flat-bottomed 24-plate at high density (HD), defined as 1.5 × 10^7^ cells/well (total volume 1.5 mL/well), in serum-free medium (CTL-Test Medium, CTL Europe GmbH, Bonn, Germany) supplemented with glutamine (2 mM), pyruvate (1 mM), penicillin, and streptomycin (100 IU/mL), at 37°C in 5% CO_2_. PBMCs were washed and cultured in CTL-Test medium at 1 × 10^5^ cells per well, in a round-bottomed 96-well plate. These cultures were then stimulated with soluble Avastin (5 μg/mL), OKT3 (5 μg/mL), TGN1412 (5 μg/mL), Campath (5 μg/mL), or IgG1 Fc Hexamer (100 μg/mL) and incubated at 37°C in 5% CO_2_. T-cell proliferation was quantified at 72 h and cytokine release was quantified 24 h post-stimulation.

### Whole Blood Assay

Blood from healthy human volunteers was collected into lithium heparin vacutainers (BD) and used within 2 h of the blood draw. Minimally-diluted blood was stimulated with either 100 μg/mL of IgG1 Fc hexamer or 10 μg/mL Campath (Genzyme). Briefly, 12.5 μL of 20x final concentration Avastin (5 μg/mL), OKT3 (5 μg/mL), TGN1412 (5 μg/mL), Campath (5 μg/mL), or IgG1 Fc Hexamer (100 μg/mL) was transferred to a 96-well round bottom tissue culture plate (Costar). 237.5 μL of whole blood was added and mixed gently by pipetting. Plates were incubated at 37°C, 5 % CO_2_, 100 % humidity for 24 h, centrifuged at 300 g for 5 min and plasma collected for cytokine analysis. Plasma not analyzed immediately was stored at −80°C until analysis.

### Flow Cytometry

1 × 10^6^ PBMCs or 100 μL of whole blood (diluted 1:2 with PBS) were stained with the appropriate fluorochrome-conjugated mAb for 30 min at 4°C and washed once. Samples were stained with anti-CD3 PerCP (clone: SK7), anti-CD56–PE (clone: HCD56), anti-CD19 APC-Cy7 (clone: HIB19), anti-CD14–Pacific Blue (clone: M5E2) and IgG1κ-FITC (clone: MOPC-21) isotype control (all from BioLegend). FcγR staining was carried out using anti-FcγRI FITC (clone: 10.1, F(ab') _2_), anti-FcγRIIa FITC (clone: E08, F(ab')_2_), anti-FcγRIIb FITC (clone: 6G11, F(ab')_2_), anti-hFcγRIIIa FITC (clone: 3G8, F(ab')_2_), and isotype control human IgG1 FITC (clone: FITC8 F(ab')_2_), (generated from published sequences in-house or sourced from BioInvent International AB). Results are shown as geometric mean fluorescent intensity (MFI) for FcγR expression on B cells (FSC-A^lo^SSC-A^lo^CD19^+^CD3^−^), NK cells (FSC-A^lo^SSC-A^lo^CD56^dim^CD3^−^, CD56^bright^CD3^+^ or CD56^hi^CD3^+^), classical monocytes (FSC-A^int^SSC-A^int^CD14^hi^), non-classical monocytes (FSC-A^int^SSC-A^int^CD14^lo^) and granulocytes (CD14^−^SSC^hi^), (see Supplementary Figure 1 for FACS gating strategy). FcγR expression levels were corrected by subtracting the geometric MFI of the corresponding isotype control staining.

Intracellular IFN-γ staining of PBMCs was carried out by culturing mAb-treated PBMCs with Golgi plug (BD Biosciences) for 24 h. Cells were stained with anti-CD4 Pacific blue (clone: SK3, BioLegend), anti-CD8 V500 (clone: RPA-T8, BD Biosciences) and anti-CD56 PE (clone: HCD56, BioLegend). PBMCs were fixed with FOXP3 Fix/Perm buffer (BioLegend) and permeabilized with FOXP3 Perm buffer before staining with anti-IFN-γ PE-Cy7 (clone: 4S.B3, eBiosciences). Samples were analyzed on a BD FACSCanto II (BD Biosciences) and data was analyzed using FlowJo Version 9.4.11 (Tree Star).

### T-Cell Proliferation Assay

PBMCs were labeled with 2 μM carboxyfluorescein succinimidyl ester (CFSE). Cells were cultured in a 24-well plate at 1 × 10^7^/mL for 24 h prior to the stimulation assays. Cells were transferred into round-bottomed 96-well plates at 1 × 10^5^ per well. On day 3, cells were labeled with anti-CD8-APC (clone: SK1, BioLegend) and anti-CD4-PE (clone C4/120: in-house), and proliferation was assessed by CFSE dilution on a FACSCalibur or FACSCanto flow cytometer (BD Biosciences). CD4^+^ and CD8^+^ T cell division is defined as a percentage of total cells excluding the parent population (first peak).

### Cytokine Determination

Supernatants from PBMC and plasma from whole blood assays were taken 24 h post-stimulation. IFN-γ, TNFα, IL-1β, and IL-6 levels were determined using the V-plex Proinflammatory Panel 1 (human) 4-plex Kit (Cat No: K15052D-2, Meso Scale Discovery) as per the manufacturer's protocol.

### Statistical Analysis

Chi-squared tests were used to compare cohorts in terms of genotype frequency and to test for Hardy-Weinberg equilibrium. Where appropriate to conform to the assumption of “Normality” and constant variance, continuous data was log-transformed prior to analysis, results back-transformed to give geometric means and cytokine release data plotted with a logarithmic axis. One-way analysis of variance (ANOVA) with *post-hoc* pairwise comparisons was used to compare donor groups with different *FCGR* alleles. Two-way ANOVA with *post-hoc* pairwise comparisons or a paired student's *t*-test were used to compare groups where the same donors were used in each group (e.g., comparing immune cells subset frequencies in donor matched whole blood and freeze-thawed PBMCs). As a large number of statistical tests have been carried out in a range of contexts, there may be an issue with multiplicity of *p*-values. Formal multiplicity adjustments have not been used, so *p*-values should be interpreted with care and within the overall scientific context. Data analysis was carried out using the Graphpad Prism version 8.0.1 software. Statistical significance defined as ^*^p < 0.05, ^**^p < 0.01 ^***^p < 0.001 and ^****^p < 0.0001 and ns = non-significant.

## Results

### Low-Affinity FcγR Gene Locus Characterization in Two Independent Cohorts

We assessed the frequency of common SNPs in the low-affinity FcγR locus in two independent cohorts using an FcγR-specific MLPA assay (Supplementary Table 1). Genotype frequencies for the combined cohorts are displayed in Table 1. Genotyping of the genes not reported to be affected by CNV, *FCGR2A* and *FCGR2B*, showed allele frequencies of 23.99% RR, 52.77% RH and 21.4% HH, and of 77.86% II, 20.66% IT and 0.37% TT, respectively. Genotypes reported for *FCGR3A, FCGR2C*, and *FCGR3B* include those with CNV. Frequencies for *FCGR3A* were 33.58% FF, 49.45% FV, and 8.86% VV; *FCGR2C* were 53.14% XX, 21.03% XQ and 2.21% QQ; and *FCGR3B* were 4.43% AA, 43.54% AB, and 31.37% BB. Reported genotypes were within Hardy-Weinberg equilibrium.

**Table 1:** Combined genotype frequencies of common low-affinity FcγR genes in the combined cohorts.

**Gene**	**SNP(s)**	**Genotype**	***N***	**%**	***p*-value**	**Chi^**2**^ test**
*FCGR2A*	rs1208724	RR	65	23.99	0.42	0.51
		RH	143	52.77		
		HH	58	21.40		
		Failed	5	1.85		
*FCGR3A*	rs396991	FF	91	33.58	0.42	0.66
		FV	134	49.45		
		VV	24	8.86		
		F	1	0.37		
		V	1	0.37		
		FFF	6	2.21		
		FFV	1	0.37		
		VVV	1	0.37		
		Failed	12	4.43		
*FCGR2C*	rs759550223	XX	144	53.14	0.48	0.5
		XQ	57	21.03		
		QQ	6	2.21		
		X	17	6.27		
		Q	9	3.32		
		XXX	25	9.23		
		XXQ	4	1.48		
		XQQ	1	0.37		
		QQQ	4	1.48		
		XXXX	1	0.37		
		Failed	3	1.11		
*FCGR3B*	HNA isoforms (rs200688856 and rs5030738)	AA	12	4.43	0.09	2.86
		AB	118	43.54		
		BB	85	31.37		
		A	8	2.95		
		B	15	5.54		
		AAA	4	1.48		
		AAB	11	4.06		
		ABB	10	3.69		
		BBBB	1	0.00		
		Failed	7	0.37		
*FCGR2B*	rs1050501	II	211	77.86	0.9	0.02
		IT	56	20.66		
		TT	1	0.37		
		Failed	3	1.11		

*HWE was calculated based on diploid genotypes within the population. A p-value >0.05 (Chi^2^>3.84) was considered to be within HWE*.

Copy gain (25.8%) across the locus was more prevalent than copy loss (20.2%). We found alterations in CNRs 1 and 2, with CNR2 the most prevalent event (Table 2). Frequencies of CNV and CNR events for each cohort are described individually in Supplementary Tables 2, 3 and for samples with available functional data in Supplementary Tables 4, 5.

**Table 2:** Copy number variation event frequencies across low-affinity FcγR genes in the combined cohorts.

**Gene**	**Gene copy**	**Total (n)**	**Total (%)**
*FCGR3A*	1	2	0.7
	2	243	90.7
	3	7	2.6
*FCGR2C*	1	26	9.7
	2	207	77.2
	3	31	11.6
	4	1	0.4
*FCGR3B*	1	23	8.6
	2	215	80.2
	3	25	9.3
	4	1	0.4
**Event**	**Gain (%)**	**Loss (%)**	**Total (%)**
**CNR1**	7 (12.1)	2 (3.4)	9 (15.5)
**CNR2**	25 (43.1)	23 (39.7)	48 (82.8)
**CNR1/2**	1 (1.7)	0 (0)	1 (1.8)

### Immune Cell Subset Frequencies and FcγR Expression on Healthy Donor PBMCs

**Figure 1:**
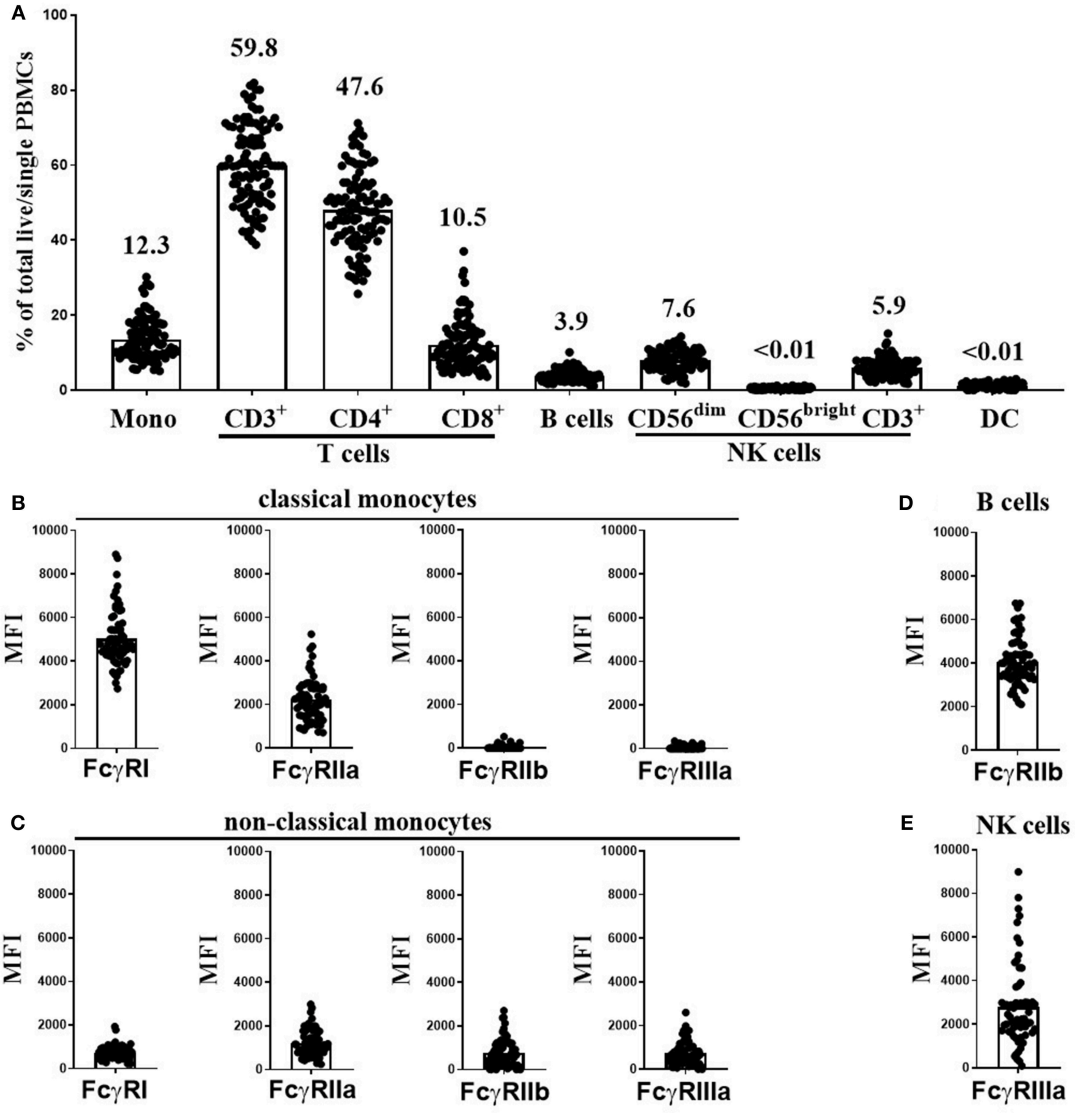
Immune cell subset frequencies and FcγR expression on PBMCs from healthy donors. Immune cell subset frequencies and FcγR expression on freeze-thawed PBMCs using flow cytometry. **(A)** Quantification of monocytes (Mono), CD3^+^, CD4^+^, and CD8^+^ T cells, B cells, CD56^dim^, CD56^bright^, and CD3^+^ NK cells and dendritic cell (DC) frequencies in freeze-thawed PBMCs from healthy human subjects (mean frequency of each cell subset stated above bar). FcγR expression on **(B)** classical, and **(C)** non-classical monocytes, **(D)** FcγRIIb expression on B cells and **(E)** FcγRIIIa expression on CD56^dim^ NK cells (*n* = 107). Each point represents a donor, bars represent group means.

Using flow cytometry (see Supplementary Figure 1 for FACS gating strategy), we assessed the cellular constituents and FcγR expression in freeze-thawed PBMC samples from 107 healthy individuals from the Southampton cohort. These donors were selected to generate a cohort with the full range of FcγR SNPs and CNV status with the potential to confer low- and high-affinity IgG binding. As expected, T cells were the most abundant cell type in these samples (median 59.8%, range 38.8–81.8%), followed by monocytes (median 12.3%, range 5.1–30.2%), CD56^dim^ NK cells (median 7.6%, range 1.8–14.3%), CD3^+^ NK cells (median 5.9%, range 1.7–15.1%) and B cells (median 3.9%, range 1.1–10.1%). CD4^+^ T cells (median 47.6%, range 25.2–71.2%) were more abundant than CD8^+^ T cells (median 10.5%, range 3.6–37%) in all donor samples. The frequencies of CD56^bright^ NK cells and dendritic cells (DCs) in these samples were <0.01% of total cells (Figure 1A).

Monocytes can be categorized into classical and non-classical subsets on the basis of CD14 high (CD14^hi^) or low (CD14^lo^) expression, respectively. FcγR expression levels on each monocyte population were assessed separately. As previously reported (31), CD14^hi^ monocytes abundantly expressed FcγRI and FcγRIIa, but were low or negative for FcγRIIb and FcγRIIIa (Figure 1B). In contrast, the less frequent CD14^lo^ non-classical monocytes expressed lower levels of FcγRI and FcγRIIa and higher levels FcγRIIb and FcγRIIIa (Figure 1C). B cells expressed high levels of FcγRIIb in comparison to non–classical monocytes but did not express any other FcγR (Figure 1D). CD56^dim^ NK cells expressed very variable levels of FcγRIIIa (Figure 1E, median MFI 2242, and range 97-8987). Finally, CD3^+^ T and CD3^+^ NK cells were negative for any cell surface FcγR expression (data not shown).

### mAb Mediated T-Cell Proliferation and Cytokine Release Using a PBMC-Based Assay Format

Pre-stored frozen PBMC samples are widely used in academia and industry, to facilitate genotyping and subsequent analysis. In the current study, PBMC samples from healthy donors, were frozen and then re-thawed for use in a PBMC-based cytokine release assay. In these assays, we opted to test the T cell-targeting antibodies, OKT3 (anti-CD3, mouse IgG2a) and TGN1412 (anti-CD28, human IgG4), renowned for inducing CRS in human subjects (5, 6). In order to establish a methodology to predict such CRS *in vitro*, Hunnig et al. developed a modified PBMC-based assay. They showed that PBMCs precultured at high density (HD), but not fresh PBMCs or whole blood, respond to TGN1412 with cytokine release *in vitro*, mimicking the proinflammatory effects observed in the clinic (8). We subsequently reported that the response to TGN1412 was a consequence of a pronounced upregulation of FcγRIIb on monocytes in the HD PBMC culture (10). Given these results and the accepted importance of FcγRs in mediating mAb effector functions, we utilized this PBMC-based HD preculture assay to assess the impact of donor FcγR genotype on T-cell proliferation and cytokine release in response to mAbs of differing functional classes which bind functionally disparate targets.

**Figure 2:**
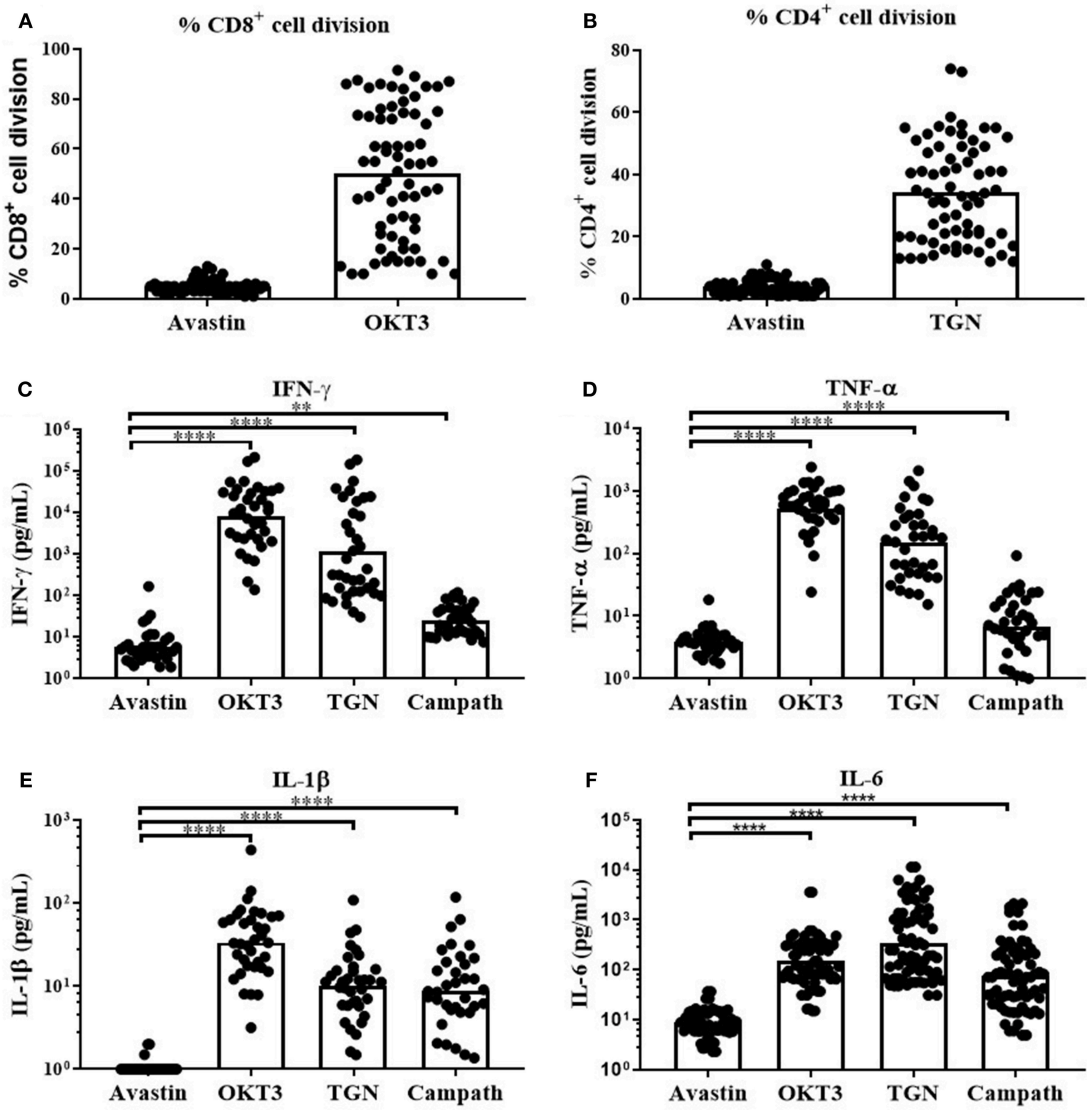
*In vitro* T-cell proliferation and cytokine release in response to mAb stimulation of freeze-thawed PBMCs. T-cell proliferation and cytokine release in response to mAb stimulation. **(A)** % CD8^+^ cell division in PBMC cultures stimulated with Avastin or OKT3 and **(B)** % CD4^+^ cell division in PBMC cultures stimulated with Avastin or TGN1412 (TGN), (*n* = 69, bars represent group means). **(C)** IFN-γ, **(D)** TNF-α, **(E)** IL-1β, and **(F)** IL-6 release by PBMCs stimulated with Avastin, OKT3, TGN1412 or Campath (*n* = 36, bars represent group geometric means). **p < 0.01 and ****p < 0.0001.

Consequently, OKT3, TGN1412 and the clinically relevant anti-CD52 (Campath, human IgG1) were used to assess the magnitude and variability of the cytokine release across our donor cohort using this assay platform. We observed a predominant but highly variable, CD8^+^ T-cell division in response to the anti-CD3 mAb (Figure 2A, range 10-92% division of total CD8^+^ cells). In contrast, TGN1412 predominantly induced CD4^+^ T-cell division (Figure 2A, range 12–74% of total CD4^+^ cells). T-cell proliferation in response to the control anti-VEGF mAb Avastin was negligible in both T cell subsets (Figures 2A,B).

We next assessed cytokine release in response to OKT3, TGN142 and Campath. All 3 mAb induced strong IFN-γ, TNF-α, IL-1β, and IL-6 responses in comparison to Avastin. Cytokine responses to the T-cell specific mAb OKT3 and TGN1412 were stronger than those observed in Campath-stimulated cultures. However, there was marked variability in the magnitude of donor responses to all three mAb treatments (Figures 2C–F). IFN-γ responses to OKT3, TGN1412 and Campath were stable over time, since stimulating PBMCs from the same donor on five separate occasions (over a 6-month period) revealed a similar response on each occasion (Supplementary Figure 2). The IFN-γ response to OKT3, TGN1412, and Campath, was significantly correlated with TNF-α, IL-1β, and IL-6 release (*R*^2^ = 0.27–0.83, *p* ≤ 0.002–0.0001 for IFN-γ vs. the other three cytokines (except IFN-γ vs. TNF-α for Campath), Supplementary Figure 3), and therefore we chose to present IFN-γ release as an exemplar read-out to assess all further cytokine responses in these assays.

**Figure 3:**
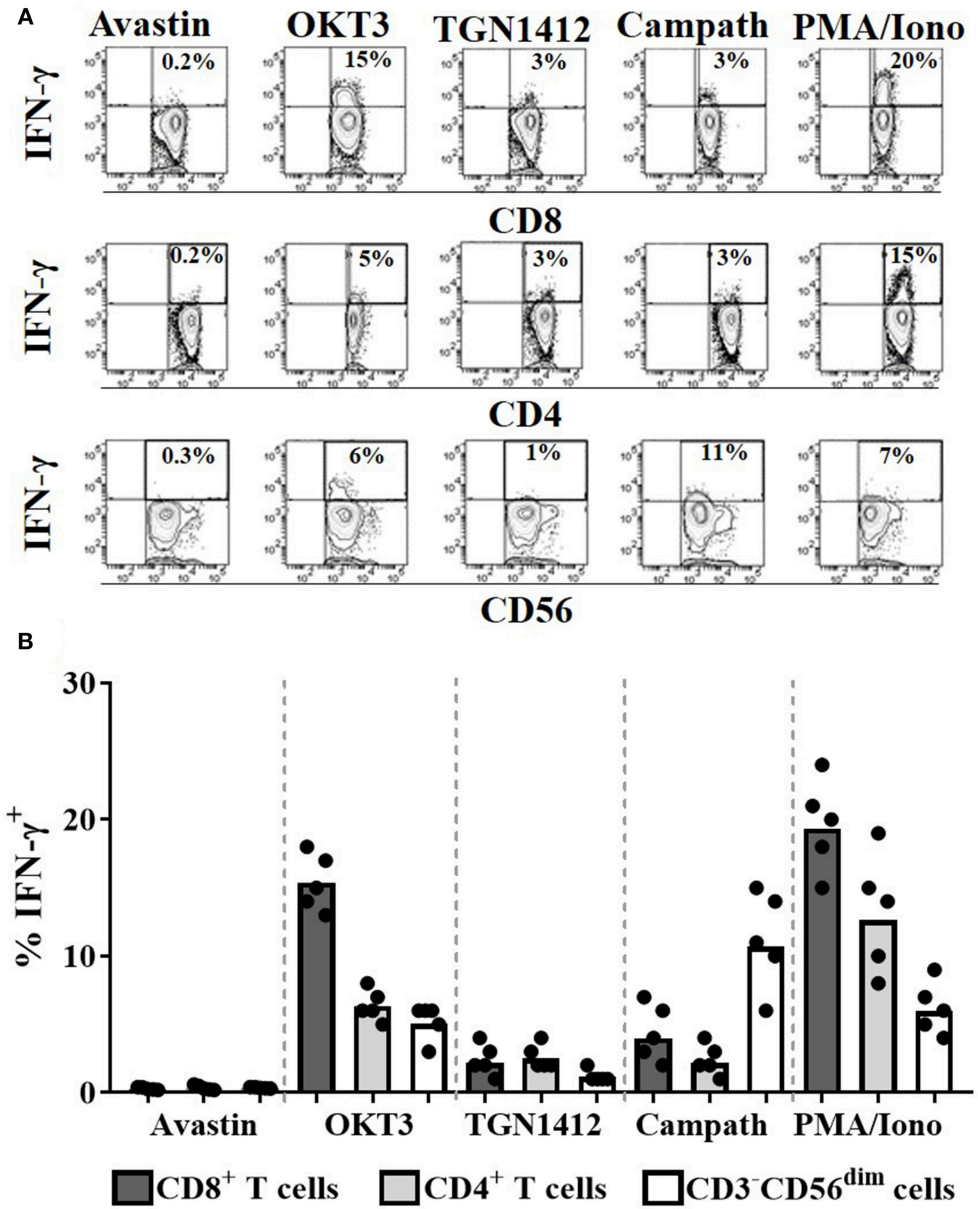
Cellular source of the IFN-γ in response to mAb stimulation. Intracellular IFN-γ staining of PBMCs stimulated for 24 h with Avastin, OKT3, TGN1412, Campath or PMA/Ionomycin (PMA/Iono). **(A)** Representative FACS contour plots of IFN-γ vs. CD8, CD4, or CD56 staining of PBMC cultures stimulated with the aforementioned treatments. **(B)** % CD8^+^ T cells, CD4^+^ T cells, and CD56^dim^ NK cells that are IFN-γ^+^ post-stimulation (*n* = 5, bars represent group means).

To assess the cell populations responsible for this IFN-γ release, we performed intracellular cytokine staining on permeabilized cells stimulated with OKT3, TGN1412, or Campath. Flow cytometry was used to identify and determine the percentage of each cell population secreting IFN-γ. The source of IFN-γ was CD8^+^ T cells in response to OKT3, both CD4^+^ and CD8^+^ T cells in response to TGN1412 and NK cells in response to Campath (Figures 3A,B). We next assessed the impact of donor FcγR genotype on the magnitude and variability of these responses observed using this HD freeze-thawed PBMC assay format.

### FcγR Genotype Does Not Significantly Impact on mAb-Mediated IFN-γ Secretion in a HD PBMC Assay Format

**Figure 4:**
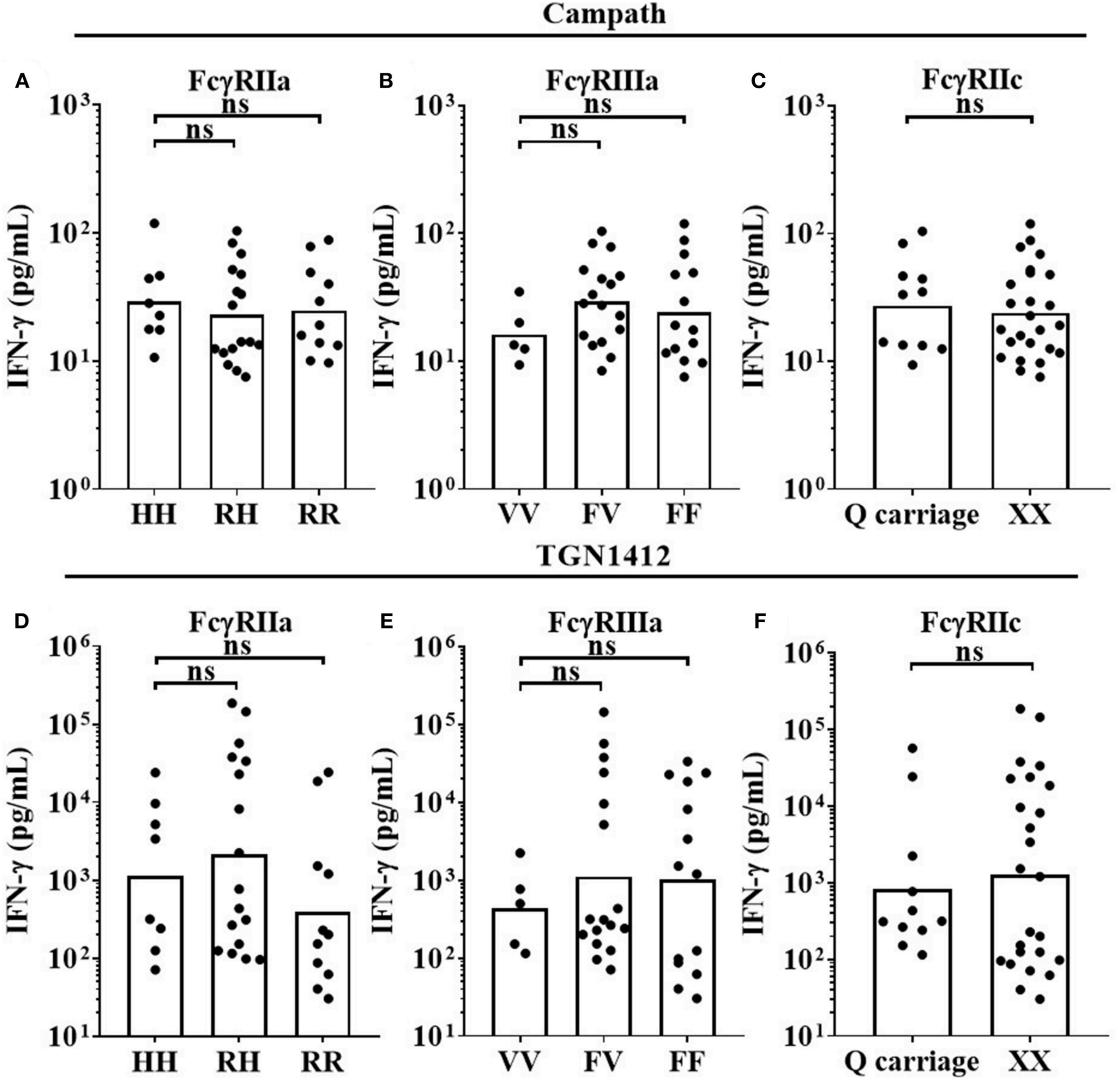
Impact of FcγR polymorphisms on mAb mediated IFN-γ release in a PBMC based assay format. Healthy donors were grouped by FcγRIIa polymorphisms; HH (*n* = 8), RH (*n* = 17) and RR (*n* = 11), FcγRIIIa polymorphisms; VV (*n* = 5), FV (*n* = 17) and FF (*n* = 14), and FcγRIIc polymorphisms; QQ/XQ (n = 11) and XX (*n* = 25). PBMCs were stimulated with **(A–C)** Campath or **(D–F)** TGN1412 and IFN-γ release was quantified 24 h post-stimulation. Each point represents a donor and bars represent group geometric means. ns = non-significant.

Three key FcγR polymorphisms have been previously associated with mAb effector capacity, defining high or low affinity receptors for FcγRIIa, FcγRIIIa and a stop codon in FcγRIIc (20–24). We therefore determined the effects of the FcγRIIa 131H/R, FcγRIIIa 158V/F, and FcγRIIc Q/X polymorphisms on IFN-γ release in response to Campath and TGN1412 using the HD PBMC-based cytokine release assay in 36 donors. When stimulating PBMCs with Campath or TGN1412, no significant association of increased IFN-γ release was observed with any of the FcγRIIa, FcγRIIIa, or FcγRIIc alleles (Figures 4A–F). Furthermore, no significant associations were observed between FcγR SNPs and magnitude of OKT3 or TGN1412 mediated T-cell proliferation (data not shown). We hypothesized that key properties of the relevant FcγR-expressing immune cell subsets may have been altered during the isolation and storing/thawing/culture of PBMCs, potentially compromising any associations between FcγR genotype and magnitude of mAb mediated IFN-γ response. To address this, we next compared the frequencies of immune cell subsets between donor matched whole blood and freeze-thawed PBMC samples.

### Freeze-Thawed PBMCs Display Altered Immune Subset Frequencies and FcγR Expression Profiles Compared to Matched Whole Blood Samples

**Figure 5:**
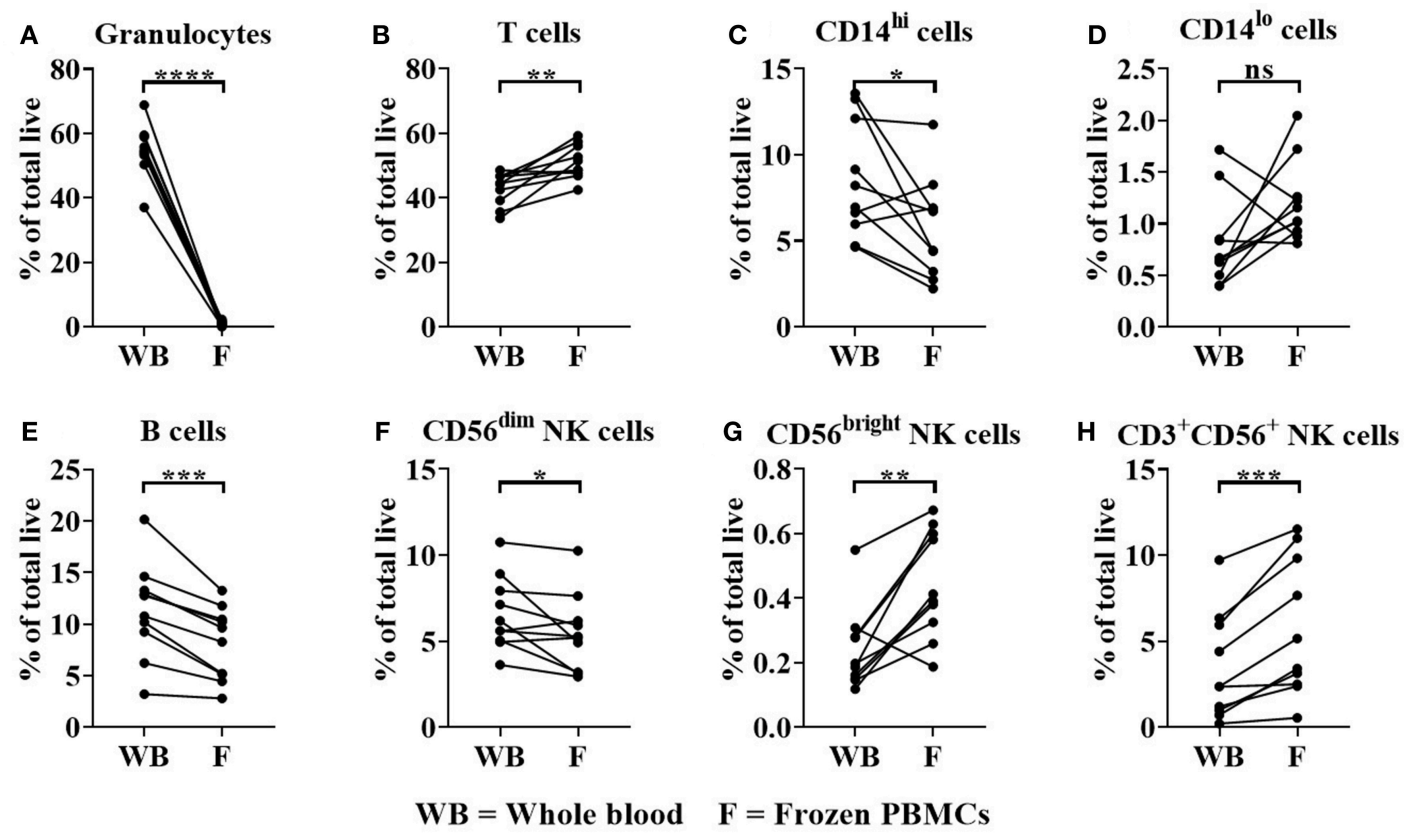
Immune cell subset frequencies in donor matched whole blood (WB) and frozen (F) PBMC samples. Immune cell subset frequencies were quantified in donor matched whole blood, fresh and frozen PBMCs using flow cytometry. Immune cell frequencies were quantified as % of total live/single cells **(A)** % Granulocytes, **(B)** T cells, **(C)** CD14^hi^ monocytes, **(D)** CD14^lo^ monocytes, **(E)** B cells, **(F)** CD3^−^CD56^dim^, **(G)** CD3^−^CD56^bright^, and **(H)** CD3^+^CD56^+^ NK cells of total live/single cells. Each point represents a donor, bars represent group means, (*n* = 10). *p < 0.05, **p < 0.01 ***p < 0.001 and ****p < 0.0001 and ns = non-significant.

As expected, whole blood had a significantly higher frequency of granulocytes in comparison to donor matched freeze-thawed (frozen) PBMC samples as a percentage of total live cells (Figure 5A); (Granulocyte median = 54.38% in whole blood compared to 1% for frozen PBMCs, *p* < 0.0001). The proportions of T cells (median = 44.41% for whole blood, 50.04% for frozen PBMCs, *p* < 0.05) were significantly enriched in frozen PBMC samples (Figure 5B). In contrast significant reductions in CD14^hi^ classical monocytes (median = 7.6% for whole blood and 5.5% for frozen PBMCs, Figure 5C), B cells (median = 11.7% for whole blood, 8.9% for frozen PBMCs, Figure 5E) and CD56^dim^ NK cells (median = 5.9% for whole blood, 5.3% for frozen PBMCs, Figure 5F) were observed when comparing whole blood to frozen PBMC samples (*p* < 0.05, *p* < 0.001 and *p* < 0.05, respectively). CD14^lo^ non-classical monocyte frequencies were not significantly altered in frozen PBMC when compared to whole blood samples (Figure 5D). The proportions of CD56^bright^ NK cells (median = 0.15% for whole blood, 0.4% for frozen PBMCs, *p* < 0.01) and CD3^+^ NK cells (median = 2.4% for whole blood, 4.3% for frozen PBMCs, *p* < 0.001) were significantly enriched in frozen PBMC samples (Figures 5G,H).

**Figure 6:**
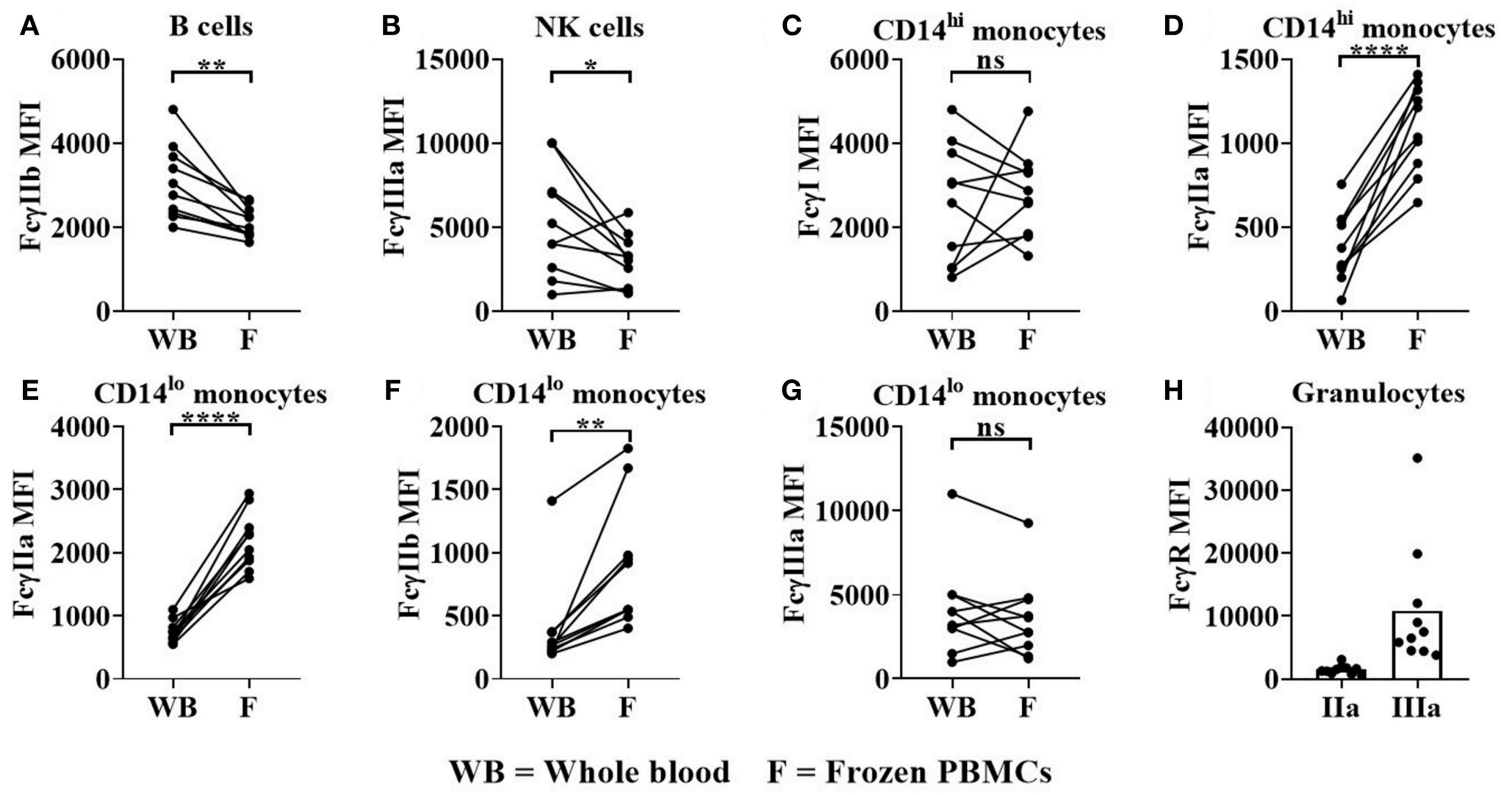
FcγR expression in donor matched whole blood (WB) and frozen (F) PBMCs. Using flow cytometry, FcγR expression was quantified on **(A)** B cells **(B)** NK cells, **(C,D)** classical monocytes, **(E–G)** non-classical monocytes and **(H)** granulocytes in donor matched WB and frozen PBMC samples. FcγRIIa (IIa) and FcγRIIIa (IIIa) expression on granulocytes was quantified in WB only. Each point represents a donor, bars represent group means, (*n* = 10). *p < 0.05, **p < 0.01, ****p < 0.0001 and ns = non-significant.

We also observed significant change in FcγR expression levels when comparing whole blood to frozen PBMCs. Significant reductions in FcγRIIb on B cells (median = 2,904 for whole blood, 2115 for frozen PBMCs, *p* < 0.01 Figure 6A) and FcγRIIIa (median = 4,618 for whole blood, 3,144 for frozen PBMCs, *p* < 0.05, Figure 6B) on NK cells were observed in frozen PBMCs compared to whole blood. FcγRI expression remained constant whereas FcγRIIa was significantly upregulated on classical monocytes in frozen PBMCs (median = 327 for whole blood, 1,124 for frozen PBMCs, *p* < 0.0001, Figure 6C). FcγRIIa (median = 701 for whole blood, 2,167 for frozen PBMCs, *p* < 0.0001) and FcγRIIb (median = 264 for whole blood, 733 for frozen PBMCs, *p* < 0.01) expression levels were both significantly upregulated on non-classical monocytes (Figures 6D,E) in frozen PBMCs, whereas FcγRIIIa expression levels remained unaltered (Figure 6G). Granulocytes (present only in whole blood), did not express FcγRI and FcγRIIb, but did express low levels of FcγRIIa and high, but variable levels of FcγRIIIB (Figure 6H).

FcγR expression on freeze-thawed PBMCs was also assessed pre- and post-HD culture. As previously reported (10), FcγRI expression on monocytes was not significantly altered (Supplementary Figure 4A). In contrast FcγRIIb was markedly increased (100-fold increase post-HD culture, *p* < 0.01), whereas increases in FcγRIIa (0.75-fold increase, *p* < 0.05) and FcγRIIIa (3-fold increase, *p* < 0.05) were less pronounced (Supplementary Figures 4B–D). FcγRIIb expression on B cells (Supplementary Figure 4E) and FcγRIIIa expression on NK cells were also not significantly modified in freeze-thawed PBMCs after HD culture (Supplementary Figure 4F).

These effects of freeze-thawing on FcγR expression and NK cell/monocyte frequencies in PBMC samples prompted us to re-assess the IFN-γ response of those therapeutics which induce cytokine release via Fc:FcγR interactions, using a whole blood assay format.

### FcγR Polymorphisms Determine the Magnitude of mAb Dependent IFN-γ Secretion in a Whole Blood Assay Format

To determine whether the impact of FcγR polymorphisms on IFN-γ release in response to Campath stimulation (an effect largely mediated by Fc:FcγR interactions) can be assessed *in vitro*, we utilized the whole blood assay format and samples from 88 UCB cohort donors. Furthermore, to restrict ourselves solely to Fc:FcγR effects, without a bias from the target receptor (e.g., CD52) we also stimulated these whole blood samples with an IgG1 Fc hexamer, which is a recombinant human IgG1 Fc construct generated by fusing the human IgG1 Fc domain to the tail-piece domain of human IgM (28). The IgG1 Fc hexamer was designed as a high-avidity FcγR blocking agent, but was demonstrated to induce high levels of pro-inflammatory cytokine release in whole blood *in vitro* assays, but not PBMC assays via a mechanism dependent on the presence of neutrophils and interactions with FcγRIIa and FcγRIIIb (29). We used this IgG1 Fc Hexamer here as a target receptor-independent mimic for ordered immune complexes which may form after mAb infusion and pose a CRS risk.

IFN-γ responses to Campath (median = 4,051 pg/ml) and the IgG1 Fc hexamer (5,588 pg/mL, *p* < 0.001) for both reagents were significantly stronger than PBS-treated controls, (11.72 pg/ml, Supplementary Figure 5A). Furthermore, there was a significant correlation (R^2^ = 0.53, *p* < 0.0001) of IFN-γ release between Campath and IgG1 Fc Hexamer treatment (Supplementary Figure 5B). Longitudinal assessment of IFN-γ release over a period of four months using repeat whole blood samples from nine heathy donors, in five separate assays, revealed stable responses from all donors to Campath and IgG1 Fc hexamer stimulation (Supplementary Figures 6A,B)—again indicating a stable donor-specific response profile, with potential value as a prognostic test.

**Figure 7:**
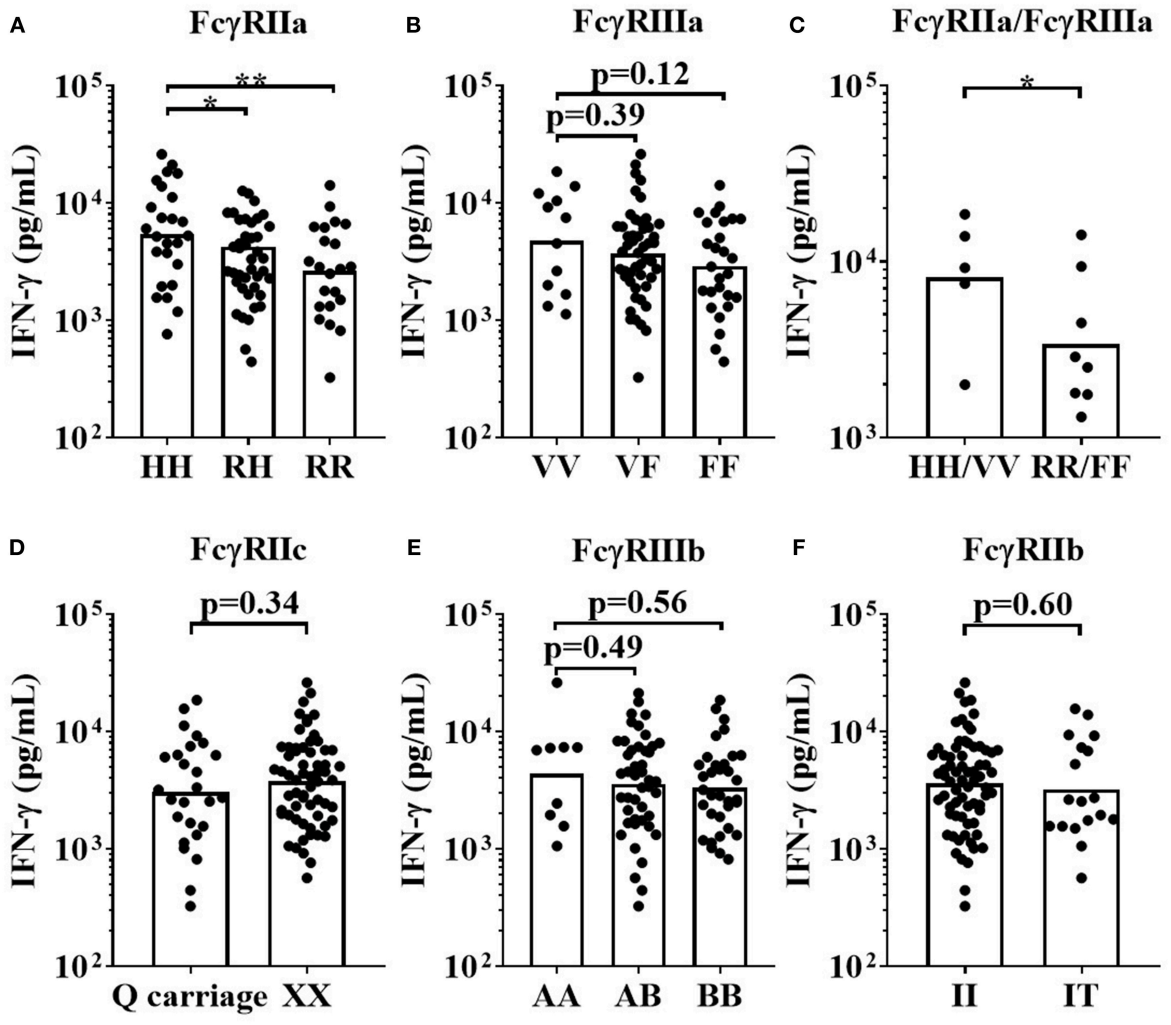
Impact of FcγR polymorphisms on Campath induced IFN-γ release in a whole blood assay format. Healthy donors were grouped by **(A)** FcγRIIa polymorphisms; HH (*n* = 25), RH (*n* = 41) and RR (*n* = 22), **(B)** FcγRIIIa polymorphisms; VV (*n* = 12), FV (*n* = 49) and FF (*n* = 27), **(C)** high affinity FcγR polymorphisms HH/VV (*n* = 5) and low affinity FcγR polymorphisms RR/FF (*n* = 8), **(D)** FcγRIIc polymorphisms; Q carriage (*n* = 26) and XX (*n*= 62), **(E)** FcγRIIIb polymorphisms; AA (*n* = 9), AB (*n* = 45) and BB (*n* = 34) and **(F)** FcγRIIb polymorphisms; II (*n* = 70) and IT (*n* = 18). Whole blood from each donor was stimulated with Campath and IFN-γ release was quantified 24 h post-stimulation. Each point represents a donor, bars represent group geometric means. *p < 0.05 and **p < 0.01.

This assay format revealed that donors homozygous for the high-affinity *FCGR2A*-131H allele mounted significantly stronger IFN-γ responses to Campath (median = 5,273 pg/mL), than individuals homozygous for the low IgG affinity *FCGR2A*-131R allele (median = 2,788 pg/mL, *p* < 0.01 when comparing HH individuals with RR individuals, Figure 7A). Heterozygous individuals (RH) elicited intermediate responses (median = 3,331 pg/mL, *p* < 0.05 when comparing HH individuals with RH individuals, Figure 7A). There were no statistically significant differences in IFN-γ release, between the high affinity homozygous *FCGR3A*-158V allele, heterozygous VF and homozygous *FCGR3A*-158F low IgG affinity donors, in response to Campath stimulation. However, this may have been due to the low numbers of donors homozygous for the high affinity *FCGR3A*-158V allele in our cohort (median; VV = 6,005 pg/mL, VF = 3,847 pg/mL and FF = 2,869 pg/mL, *p* = 0.26 when comparing VV vs. FF donor responses, Figure 7B). Significantly greater IFN-γ release was observed in response to Campath stimulation amongst donors homozygous for both high affinity *FCGR2A*-131H and *FCGR3A*-158V SNPs (median = 9204 pg/mL) in comparison to donors homozygous for the low IgG affinity *FCGR2A*-131R and *FCGR3A*-158F alleles (median = 2,684 pg/mL, *p* < 0.05, Figure 7C). When comparing *FCGR2C*-57Q SNP-carrying donors, who are predicted to express this additional activatory FcγR on NK cells, with *FCGR2C*-57X homozygous donors who are FcγRIIc negative, no significant associations with IFN-γ release were observed (*p* = 0.34, when comparing QQ/QX with XX donors, Figure 7D). Furthermore, no significant associations between the *FCGR3B* and *FCGR2B* SNPs and Campath induced IFN-γ release were revealed using this assay format (Figures 7E,F).

**Figure 8:**
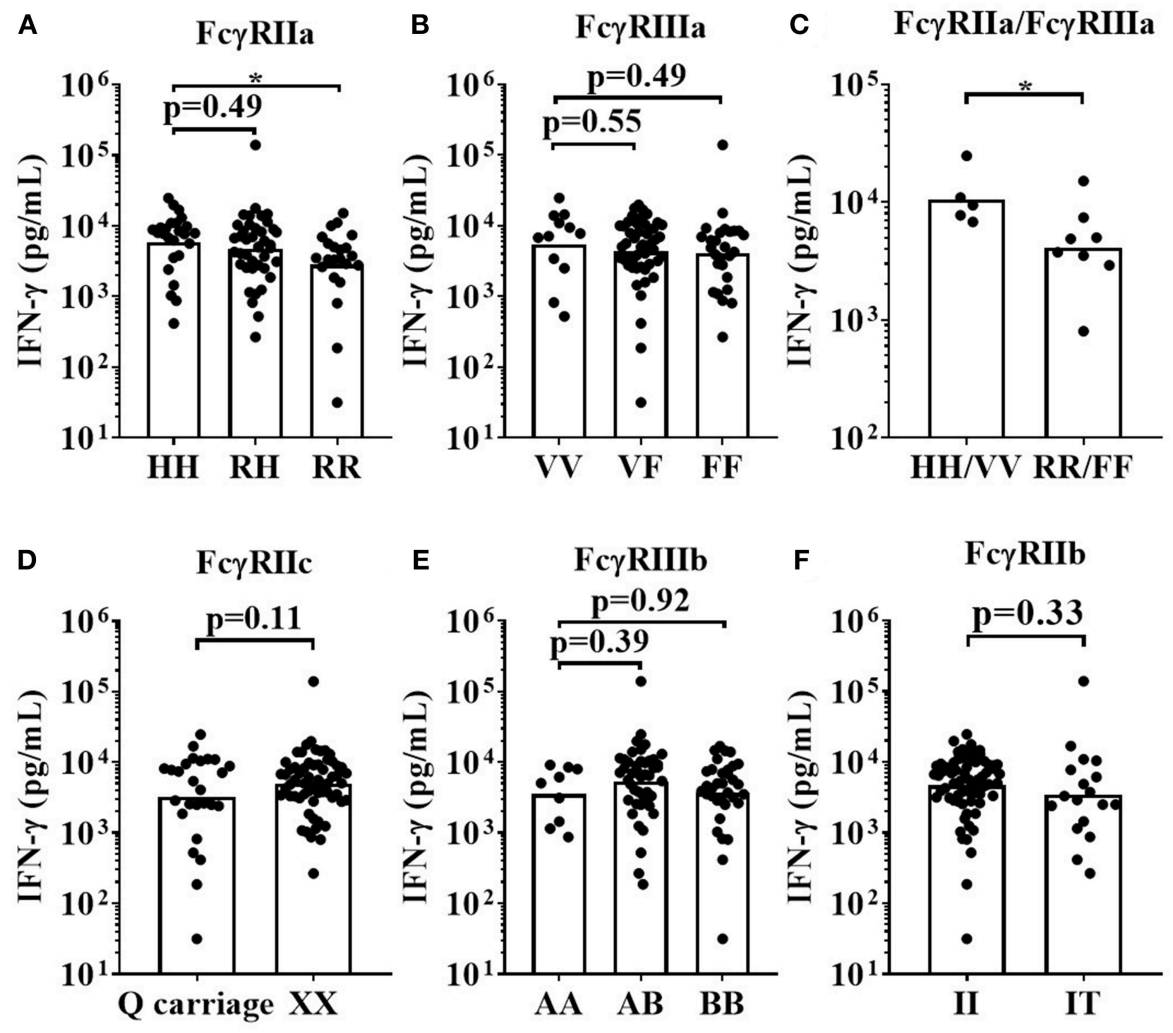
Impact of FcγR polymorphisms on IFN-γ release induced by IgG1 Fc Hexamer stimulation in a whole blood assay format. Healthy donors were grouped by; **(A)** FcγRIIa polymorphisms; HH (*n* = 25), R/H (*n* = 41), and RR (*n* = 22), **(B)** FcγRIIIa polymorphisms; VV (*n* = 12), FV (*n* = 49) and FF (*n* = 27), **(C)** high affinity FcγR polymorphisms HH/VV (*n* = 5) and low affinity FcγR polymorphisms RR/FF (*n* = 8), **(D)** FcγRIIc polymorphisms; Q carriage (*n* = 26) and XX (*n* = 62), **(E)** FcγRIIIb polymorphisms; AA (*n* = 9), AB (*n* = 45) and BB (*n* = 34) and **(F)** FcγRIIb polymorphisms; II (*n* = 70) and IT (*n* = 18). Whole blood from each donor was stimulated with IgG1 Fc Hexamer and IFN-γ release was quantified 24 h post-stimulation. Each point represents a donor, bars represent group geometric means. *p < 0.05.

For the IgG1 Fc hexamer, *FCGR2A*-131H homozygous high affinity donors mounted significantly stronger IFN-γ responses (median = 7,867 pg/mL) when compared to *FCGR2A*-131R low affinity homozygous donors (median = 3,470 pg/mL, *p* < 0.05). The *FCGR2A*-131RH heterozygous donors mounted an intermediate response (median = 5,066 pg/mL, Figure 8A). There were no statistically significant differences in IFN-γ release between the homozygous high affinity *FCGR3A*-158V allele, heterozygous VF and homozygous *FCGR3A*-158F low IgG affinity donors, in response to IgG1 Fc Hexamer stimulation. (median IFN-γ responses (pg/mL); VV = 7441, VF = 5,066 and FF = 5,443.14, Figure 8B). IFN-γ release in response to IgG1 Fc hexamer amongst donors homozygous for both the high affinity *FCGR2A*-131H and *FCGR3A*-158V alleles were significantly elevated compared to donors homozygous for the low IgG affinity alleles (median for HH/VV = 9,472 and RR/FF = 4,324 pg/mL, *p* < 0.05, Figure 8C). No significant associations between the *FCGR2C, FCGR3B* and *FCGR2B* SNPs and IgG1 Fc hexamer induced IFN-γ release were observed using this assay format (Figures 8D–F). Assessing the effects of *FCGR3A, FCGR2C*, and *FCGR3B* gene CNV with FcγR expression on immune cells or mAb mediated cytokine release was not possible in the current study due to the limited size of donor cohorts and relative rarity of gene CNV >2.

Finally, we stimulated matched whole blood and precultured freeze-thawed PBMC cultures with OKT3, TGN1412, Campath and the IgG1 Fc Hexamer, to directly compare both assay formats and modified IFN-γ release amongst five high IgG affinity *FCGR2A*-131H homozygous donors compared to five low IgG affinity *FCGR2A*-131R homozygous donors. A trend toward increased IFN-γ release was observed in the *FCGR2A*-131H homozygous donors. However, with such small donor numbers a clear statistically significant relationship could not be confirmed using either assay format (data not shown). Altogether, these results demonstrate the importance of donor assay format and sufficient sample numbers when determining the impact of *FCGR2A* and *FCGR3A* polymorphisms on the magnitude of the IFN-γ response elicited by antibodies and IgG constructs with CRS-inducing potential.

## Discussion

Beyond their effects resulting from specific binding of cell surface antigens, mAb possess additional biological activity mediated through their Fc:FcγR interactions which are typically critical for efficacious antibody immunotherapy in patients. mAb interactions with FcγRIIa and FcγRIIIa mediate antibody dependent cellular cytotoxicity (ADCC) and antibody dependent cellular phagocytosis (ADCP) by tumor targeting mAb such as Rituximab, Herceptin and Campath. SNPs in *FCGR2A* and *FCGR3A* genes influencing mAb affinity for FcγR have previously been shown to modify antibody immunotherapy in cancer patients (32). Here we report that whole blood assays are potentially more sensitive for hazard identification of mAb mediated cytokine release, as well as the assessment of the impact of *FCGR2A* and *FCGR3A* SNPs on the magnitude of this cytokine release *in vitro*.

MLPA remains the current gold standard assay for comprehensive FcγR genotyping. Using PBMC samples from healthy donors, we observed considerable variability across the FcγR locus in the form of SNPs and CNV, with homology between FcγR genes further complicating analysis as a result of the ancestral segmental duplication (33, 34). SNP frequencies described in this study are in line with others (35, 36) and while the reported SNP frequencies are within Hardy-Weinberg equilibrium, it is not optimized for loci with CNV. CNV represents a significant source of genetic diversity and can affect the function of FcγR gene products. Alterations in copy number of FcγR genes have reported gene dosage effects on protein expression (27, 37). CNV has been described in *FCGR3A, FCGR2C*, and *FCGR3B* (30, 35, 37), with rare events reported to affect *FCGR2B* (34). Regions of copy number alteration, CNR1-4, encompassing multiple genes in the locus have been described (17). As previously described (17, 35), CNV of *FCGR3A* is rare (3.6% of individuals), with alterations affecting *FCGR2C* and *FCGR3A* the most common CNV events. To date, these CNV have not been associated with mAb-mediated effects in the clinic, perhaps due to their relative rarity, leading to insufficient statistical power. Similar deficiencies were observed here in our study, with several 100 donors being required to study the impacts of CNV comprehensively. While many studies have reported associations between the high affinity *FCGR2A* and *FCGR3A* alleles and greater mAb efficacy in numerous cancers (20, 23), others have not observed such associations (38, 39), perhaps due to differing and complex biological backgrounds. We postulated that FcγR genotypes may correlate with *in vitro* IFN-γ responses to mAb stimulation and further enable prediction of CRS risk in the clinic.

In recent years, the limited value of rodent models for predicting treatment responses in humans set in motion intensive research to establish *in vitro* assays using human PBMCs; for example to predict the magnitude of cytokine release induced by therapeutic mAb (40). Assessment of mAb function and toxicity *in vitro* often utilizes banked frozen PBMC samples in both commercial pharmaceutical and academic settings. We previously used a PBMC-based assay format in which freeze-thawed PBMCs are first cultured at high density prior to stimulation with mAb. The high density preculture step promotes PBMC sensitivity to TGN1412 which otherwise elicits no immune cell activation in fresh untouched PBMC cultures (8, 10). Thus using this assay format which allows for the assessment of a CRS-inducing mAb (TGN1412), we sought to determine the impact of FcγR SNPs on mAb-induced cytokine release. Although assays were reproducible and stable per donor over time (indicating an inherent factor underpinning the level of response), no significant impact of *FCGR2A, FCGR2C*, or *FCGR3A* polymorphisms was observed with any mAb with respect to the magnitude of cytokine release (Figure 4). Larger cohorts would be required to accurately study the impact of CNV at the low-affinity locus given the low-frequency of events in *FCGR3A*, for example, whose gene product, FcγRIIIa, is an important mediator of NK cell-mediated ADCC.

This prompted a detailed comparison of immune cell subset frequencies and FcγR expression in whole blood and previously frozen PBMC samples. Significant reductions in monocytes, NK cells (CD3^−^CD56^dim^ cells) and FcγRIIIa expression on the latter were observed in freeze-thawed PBMCs relative to whole blood (Figures 5C,F, 6B). These observations were in concordance with previous studies reporting reduction in FcγRIIIa positive NK cells in freeze-thawed PBMC samples (41). In addition, FcγRIIa expression was significantly increased on monocytes in PBMC samples (Figure 6D,E), however, monocyte frequencies were reduced (Figures 5C), further impacting the likelihood of observing any *FCGR2A* SNP association with mAb-mediated cytokine release. The absence of FcγR-bearing neutrophils, platelets, donor IgG and complement proteins from serum in PBMC samples further justifies the utility of whole blood assays when determining the effects of FcγR SNPs on mAb-mediated cytokine release.

In the current study minimally diluted whole blood (95% blood / 5% mAb diluent) combined with aqueous mAb presentation was shown to be a useful and promising format, with only minimal sample and mAb manipulation aiming to preserve the natural peripheral blood molecular and cellular composition. We used this system to test Campath which binds CD52, a cell surface membrane antigen abundantly expressed on the surface of B cells, T cells, and monocytes (42). Campath triggering of FcγRIIIa on NK cells directly leads to IFN-γ release (12). We also tested an IgG1 Fc Hexamer construct which interacts with FcγRs, not target antigen. We have previously reported that cytokine release associated with this construct is primarily via interaction with FcγRIIa and FcγRIIIb and dependent on the presence of neutrophils (28, 29). Thus, both Campath and the IgG1 Fc Hexamer were suitable candidates for the assessment of the impact of FcγR SNPs on cytokine release in this assay format.

FcγRIIa is a monomeric receptor possessing an ITAM in its intracellular domain. It is the most broadly distributed FcγR, being expressed on monocytes, macrophages, platelets, and neutrophils and also in a soluble form (FcγRIIa2), (43). Our quantification of FcγRIIa expression in whole blood confirmed expression is restricted to monocytes and neutrophils (Figure 6). IgG triggering of FcγRIIa-mediated ITAM signaling results in cellular activation, phagocytosis, oxidative burst and the production of pro-inflammatory cytokines by monocytes and neutrophils (44). In whole blood, we observed a significantly elevated IFN-γ release in response to Campath and IgG1 Fc Hexamer stimulation amongst *FCGR2A*-131H/H donors compared to R/R donors. As Campath primarily stimulates cytokine release by triggering FcγRIIIa on NK cells (12), it was unexpected to observe a significant association with the *FCGR2A*-131H allele (Figure 7A). This enhanced IFN-γ release amongst the *FCGR2A*-131H homozygous donors may therefore be an indirect consequence of Campath triggered FcγRIIa activation on monocytes and neutrophils leading to pro-inflammatory cytokine release in these cell types, that then activates NK cells to secrete IFN-γ (45). We have previously demonstrated that the IgG1 Fc hexamer stimulates IFN-γ production in whole blood in a neutrophil-dependent manner, in contrast to the response to Campath which was not affected by depletion of neutrophils from whole blood (29). Isolated neutrophils have also been shown to be capable of producing pro-inflammatory cytokines (29, 46) and TLR-independent neutrophil-derived IFN-γ is important for host resistance to intracellular pathogens (28), emphasizing the importance of maintaining the presence of these FcγR bearing cells in *in vitro* cytokine release assays, especially when testing reagents with the potential to form immune complexes. Neutrophils express both FcγRIIa and FcγRIIIb and this study, along with our previous data, suggests both receptors are important in this immune-complex induced cytokine response and that polymorphisms in FcγRIIa in particular may modulate this. This is in agreement with earlier studies indicating a complex interplay between FcγRIIa and FcγRIIIb haplotype and sensitivity of neutrophils to IgG-induced respiratory burst (47).

FcγRIIIa is a type I transmembrane receptor and signals via its association with the ITAM-expressing FcRγ chain, encoded by the *FCER1G* gene (48). In whole peripheral blood its expression is largely restricted to CD3^−^CD56^dim^ NK cells, and non-classical monocytes (Figures 6B,C). Additionally, FcγRIIIa is also abundantly expressed on macrophages (not present in whole blood) as well as on tumor-associated macrophages (49). FcγRIIIa has been reported to be the most potent activating receptor on freshly isolated peripheral blood NK cells, able to elicit potent ADCC and cytokine production in response to Campath treatment (50). Although not significant, we observed enhanced IFN-γ responses to Campath and IgG1 Fc hexamer stimulated whole blood cultures sourced FCGR3A-158V/V donors relative to *FCGR3A*-158V/F and *FCGR3A*-158F/F donors. Given the lower frequency of the *FCGR3A*-158V/V genotype in Western European populations (<10%), large sample size is essential for these studies to achieve statistically significant associations with mAb mediated cytokine release. In the current study only 12/88 donors (UCB cohort) possessed the *FCGR3A*-158V/V genotype, likely explaining the lack of statistical significance.

In the whole blood assay format, mAb-mediated effector functions are profoundly influenced by simultaneous mAb interactions with FcγRIIa, FcγRIIIa, FcγRIIb, and FcγRIIIb. To partially address the impact of IgG Fc interaction with more than one FcγR species, we analyzed a subset of donors homozygous for high or low affinity *FCGR2A*-131 and *FCGR3A*-158 alleles. The HH/VV donors had a 4-fold higher IFN-γ response to Campath and a 2-fold higher response to IgG1 Fc Hexamer, in comparison to the RR/FF donors. Encouragingly, these significant differences were observed with a relatively low number of donors and were again stable over time (indicating a stable donor-specific response profile), with potential value for development of prognostic tests. However, greater donor numbers are required for sufficient statistical powering for other associations, especially when also taking into account the low frequency of certain SNPs and large variability of cytokine responses to mAb stimulation of whole blood cultures. Based upon power calculations on our data to date, we recommend ≥20 donors for each FcγR SNP for assessing associations of FcγR genotype with mAb-mediated cytokine release hazard identification. This gives more than 80% power to detect a 3-fold difference between groups, using the whole blood IFN-γ release assay (Supplementary Figure 7).

Using the whole blood assay format, we did not observe statistically significant associations between *FCGR2C, FCGR2B*, or *FCGR3B* SNPs with the magnitude of Campath or IgG1 Fc hexamer-mediated cytokine release. FcγRIIc expression has been reported on NK cells, however, using flow cytometry we observed negligible or no FcγRIIc expression on NK cells, in >100 PBMC or whole blood samples (manuscript in preparation). This may explain the lack of significant association of *FCGR2C* SNPs with the magnitude of cytokine release. In whole blood samples, FcγRIIb expression is almost entirely restricted to B cells, which are unlikely to contribute to Campath or IgG1 Fc Hexamer induced IFN-γ release. Furthermore, it is worth recollecting that FcγRIIb is an ITIM-signaling inhibitory receptor, more likely to restrict cytokine release mediated by ITAM signaling on cell types co-expressing activatory and inhibitory FcγR. In addition, the I232T *FCGR2B* SNP leading to lack of inhibitory signaling, is extremely rare in Caucasian populations and so would require an extremely large cohort to study (26). Although FcγRIIIb has been reported to play a role in IgG1 Fc Hexamer induced cytokine release (29), we observed large variability in the expression levels of this receptor on neutrophils (MFI range 3771-35100) between donor samples. This may have compromised observing significant associations of *FCGR3B* SNPs with the extent of cytokine release in the IgG1 Fc Hexamer treated samples.

In summary, while there is considerable variability in the magnitude of cytokine responses elicited by cytokine storm-inducing IgG1 antibodies and Fc constructs in the whole blood assay format, key cell populations such as NK cells, monocytes and neutrophils remain intact and express FcγR at physiological levels. Our findings suggest that high-throughput genotyping combined with whole blood assays may be a powerful pharmacogenetic approach to predict both mAb therapy outcome and hazard identification but requires sufficient donors of each FcγR genotype if these associations are sought.

## Author Contributions

KH, CH, TR, KL, and JMS performed experiments. KH, CH, and JS performed statistical analyses. KH, CH, TR, MG, JCS, and MC designed experiments. KH and CH wrote the manuscript with contributions from TR, DH, and MC. All authors contributed to manuscript revision and read and approved the submitted version.

### Conflict of Interest Statement

MC is a retained consultant for BioInvent International and has performed educational and advisory roles for Baxalta and Boehringer Ingleheim. He has received research funding from Roche, Gilead and GSK. TR, PB, JS, RC, and DH are employees of UCB Pharma. The remaining authors declare that the research was conducted in the absence of any commercial or financial relationships that could be construed as a potential conflict of interest.
