# Supplementary material for: Impact of Human FcγR Gene Polymorphisms on IgG-Triggered Cytokine Release: Critical Importance of Cell Assay Format
Source: Front Immunol. 2019 Mar 7;10:390. doi: 10.3389/fimmu.2019.00390 (PMC6417454; doi:10.3389/fimmu.2019.00390)
Supplement: Supplementary file 1 [file Data_Sheet_1.docx]

**Supplementary information**

**Impact of human FcγR gene polymorphisms on IgG-triggered cytokine release: Critical importance of cell assay format**

**Authors:** Khiyam Hussain^†1^, Chantal E. Hargreaves^†1,2,3^, Tania F. Rowley^4^, Joshua M Sopp^1^, Kate V. Latham^3^, Pallavi Bhatta^4^, John Sherington^4^, Rona M. Cutler^4^, David P Humphreys^4^, Martin J. Glennie^1^, Jonathan C. Strefford^3^, Mark S. Cragg^1*^

**Supplementary Table 1: Genotype frequency data of individual cohorts**

|  | | | **Southampton cohort** | | | |  | **UCB cohort** | | | |
| --- | --- | --- | --- | --- | --- | --- | --- | --- | --- | --- | --- |
| **Gene** | **SNP(s)** | **Genotype** | **N** | **%** | **p-value** | **Chi^2^ test** |  | **N** | **%** | **p-value** | **Chi^2^ test** |
| *FCGR2A* | rs1208724 | RR | 42 | 23.6 | 0.13 | 2.24 |  | 23 | 24.73 | 0.52 | 0.42 |
|  |  | RH | 101 | 56.74 |  |  |  | 42 | 45.16 |  |  |
|  |  | HH | 33 | 18.54 |  |  |  | 25 | 26.88 |  |  |
|  |  | Failed | 2 | 1.12 |  |  |  | 3 | 3.23 |  |  |
| *FCGR3A* | rs396991 | FF | 64 | 35.96 | 0.1 | 2.47 |  | 27 | 29.03 | 0.42 | 0.66 |
|  |  | FV | 85 | 47.75 |  |  |  | 49 | 52.69 |  |  |
|  |  | VV | 13 | 7.30 |  |  |  | 11 | 11.83 |  |  |
|  |  | F | 1 | 0.56 |  |  |  | 0 | 0.00 |  |  |
|  |  | V | 1 | 0.56 |  |  |  | 0 | 0.00 |  |  |
|  |  | FFF | 4 | 2.25 |  |  |  | 2 | 2.15 |  |  |
|  |  | FFV | 1 | 0.56 |  |  |  | 0 | 0.00 |  |  |
|  |  | VVV | 0 | 0.00 |  |  |  | 1 | 1.08 |  |  |
|  |  | Failed | 9 | 5.06 |  |  |  | 3 | 3.23 |  |  |
| *FCGR2C* | rs759550223 | XX | 94 | 52.81 | 0.78 | 0.08 |  | 50 | 53.76 | 0.5 | 0.5 |
|  |  | XQ | 38 | 21.35 |  |  |  | 19 | 20.43 |  |  |
|  |  | QQ | 3 | 1.69 |  |  |  | 3 | 3.23 |  |  |
|  |  | X | 12 | 6.74 |  |  |  | 5 | 5.38 |  |  |
|  |  | Q | 7 | 3.93 |  |  |  | 2 | 2.15 |  |  |
|  |  | XXX | 16 | 8.99 |  |  |  | 9 | 9.68 |  |  |
|  |  | XXQ | 2 | 1.12 |  |  |  | 2 | 2.15 |  |  |
|  |  | XQQ | 1 | 0.56 |  |  |  | 0 | 0.00 |  |  |
|  |  | QQQ | 4 | 2.25 |  |  |  | 0 | 0.00 |  |  |
|  |  | XXXX | 1 | 0.56 |  |  |  | 0 | 0.00 |  |  |
|  |  | Failed | 0 | 0 |  |  |  | 3 | 3.23 |  |  |
| *FCGR3B* | HNA isoforms  (rs200688856 and rs5030738) | AA | 7 | 3.93 | 0.02 | 5.67 |  | 5 | 5.38 | 0.09 | 2.86 |
|  |  | AB | 79 | 44.38 |  |  |  | 39 | 41.94 |  |  |
|  |  | BB | 55 | 30.9 |  |  |  | 30 | 32.26 |  |  |
|  |  | A | 4 | 2.25 |  |  |  | 4 | 4.30 |  |  |
|  |  | B | 11 | 6.18 |  |  |  | 4 | 4.30 |  |  |
|  |  | AAA | 0 | 0.00 |  |  |  | 4 | 4.30 |  |  |
|  |  | AAB | 7 | 3.93 |  |  |  | 4 | 4.30 |  |  |
|  |  | ABB | 10 | 5.62 |  |  |  | 0 | 0.00 |  |  |
|  |  | BBB | 0 | 0.00 |  |  |  | 4 | 0.00 |  |  |
|  |  | BBBB | 1 | 0.56 |  |  |  | 0 | 0.00 |  |  |
|  |  | Failed | 4 | 2.25 |  |  |  | 3 | 3.23 |  |  |
| *FCGR2B* | rs1050501 | II | 140 | 78.65 | 0.23 | 1.43 |  | 71 | 76.34 | 0.9 | 0.02 |
|  |  | IT | 38 | 21.35 |  |  |  | 18 | 19.35 |  |  |
|  |  | TT | 0 | 0.00 |  |  |  | 1 | 1.08 |  |  |
|  |  | Failed | 0 | 0 |  |  |  | 3 | 3.23 |  |  |

HWE was calculated on diploid genotypes within a population. . A p-value > 0.05 (Chi^2^ > 3.84) was considered to be within HWE.

**Supplementary Table 2: CNV frequency data of individual cohorts**

|  | | | **Southampton cohort** | | **UCB cohort** | | |
| --- | --- | --- | --- | --- | --- | --- | --- |
| **Gene** | **Copy number** | **N** | | **%** | **N** | **%** |  |
| *FCGR3A* | 1 | 2 | | 1.1 | 0 | 0.0 |  |
|  | 2 | 154 | | 86.5 | 87 | 96.7 |  |
|  | 3 | 5 | | 2.8 | 2 | 2.2 |  |
| *FCGR2C* | 1 | 19 | | 10.7 | 7 | 7.8 |  |
|  | 2 | 135 | | 75.8 | 72 | 80.0 |  |
|  | 3 | 20 | | 11.2 | 11 | 12.2 |  |
|  | 4 | 1 | | 0.6 | 0 | 0.0 |  |
| *FCGR3B* | 1 | 15 | | 8.4 | 8 | 8.9 |  |
|  | 2 | 141 | | 79.2 | 74 | 82.2 |  |
|  | 3 | 17 | | 9.6 | 8 | 8.9 |  |

**Supplementary Table 3: Copy number region frequencies in the low-affinity FcγR locus in individual cohorts**

|  | | **Southampton cohort** | **UCB cohort** |
| --- | --- | --- | --- |
| **CNR** | **Change** | **N (%)** | **N (%)** |
| 1 | Gain | 5 (12.5) | 7 (12.1) |
|  | Loss | 2 (5) | 2 (3.4) |
| 2 | Gain | 17 (42.5) | 23 (39.7) |
|  | Loss | 15 (37.5) | 0 (0) |
| 1/2 | Gain | 1 (2.5) | 0 (0) |
|  | Loss | 0 (0) | 0 (0) |

**Supplementary Table 4: SNP frequency of Southampton donors with available functional data**

|  | | | **Proliferation cohort** | | | | **Cytokine cohort** | | | | **Phenotype cohort** | | | |
| --- | --- | --- | --- | --- | --- | --- | --- | --- | --- | --- | --- | --- | --- | --- |
| **Gene** | **SNP(s)** | **Genotype** | **N** | **%** | **p-value** | **Chi^2^ test** | **N** | **%** | **p-value** | **Chi^2^ test** | **N** | **%** | **p-value** | **Chi^2^ test** |
| *FCGR2A* | rs1208724 | RR | 19 | 34.55 | 0.48 | 0.5 | 11 | 30.56 | 0.62 | 0.24 | 18 | 27.69 | 0.85 | 0.04 |
|  |  | RH | 25 | 45.45 |  |  | 17 | 47.22 |  |  | 33 | 50.77 |  |  |
|  |  | HH | 11 | 20.00 |  |  | 8 | 22.22 |  |  | 14 | 21.54 |  |  |
| *FCGR3A* | rs396991 | FF | 16 | 29.09 | 0.63 | 0.23 | 12 | 33.33 | 0.24 | 1.38 | 18 | 27.69 | 0.23 | 1.45 |
|  |  | FV | 24 | 43.64 |  |  | 18 | 50.00 |  |  | 33 | 50.77 |  |  |
|  |  | VV | 11 | 20.00 |  |  | 4 | 11.11 |  |  | 9 | 13.85 |  |  |
|  |  | F | 0 | 0.00 |  |  | 0 | 0.00 |  |  | 1 | 1.54 |  |  |
|  |  | V | 0 | 0.00 |  |  | 0 | 0.00 |  |  | 0 | 0.00 |  |  |
|  |  | FFF | 3 | 5.45 |  |  | 2 | 5.56 |  |  | 3 | 4.62 |  |  |
|  |  | FFV | 1 | 1.82 |  |  | 0 | 0.00 |  |  | 1 | 1.54 |  |  |
|  |  | FVV | 0 | 0.00 |  |  | 0 | 0.00 |  |  | 0 | 0.00 |  |  |
|  |  | VVV | 0 | 0.00 |  |  | 0 | 0.00 |  |  | 0 | 0.00 |  |  |
| *FCGR2C* | rs759550223 | XX | 25 | 45.45 | 0.002 | 9.54 | 18 | 50.00 | 0.43 | 0.61 | 39 | 60.00 | 0.005 | 7.8 |
|  |  | XQ | 6 | 10.91 |  |  | 4 | 11.11 |  |  | 6 | 9.23 |  |  |
|  |  | QQ | 3 | 5.45 |  |  | 0 | 0.00 |  |  | 2 | 3.08 |  |  |
|  |  | X | 4 | 7.27 |  |  | 3 | 8.33 |  |  | 4 | 6.15 |  |  |
|  |  | Q | 5 | 9.09 |  |  | 4 | 11.11 |  |  | 5 | 7.69 |  |  |
|  |  | XXX | 7 | 12.73 |  |  | 3 | 8.33 |  |  | 4 | 6.15 |  |  |
|  |  | XXQ | 0 | 0.00 |  |  | 0 | 0.00 |  |  | 0 | 0.00 |  |  |
|  |  | XQQ | 2 | 3.64 |  |  | 1 | 2.78 |  |  | 2 | 3.08 |  |  |
|  |  | QQQ | 2 | 3.64 |  |  | 2 | 5.56 |  |  | 2 | 3.08 |  |  |
|  |  | XXXX | 0 | 0.00 |  |  | 0 | 0.00 |  |  | 0 | 0.00 |  |  |
| *FCGR3B* | HNA isoforms  (rs200688856 and rs5030738) | AA | 0 | 0.00 | 0.002 | 9.23 | 1 | 2.78 | 0.11 | 2.52 | 3 | 4.62 | 0.12 | 2.43 |
|  |  | AB | 20 | 36.36 |  |  | 12 | 33.33 |  |  | 25 | 38.46 |  |  |
|  |  | BB | 17 | 30.91 |  |  | 12 | 33.33 |  |  | 21 | 32.31 |  |  |
|  |  | A | 2 | 3.64 |  |  | 2 | 5.56 |  |  | 4 | 6.15 |  |  |
|  |  | B | 6 | 10.91 |  |  | 3 | 8.33 |  |  | 4 | 6.15 |  |  |
|  |  | AAA | 0 | 0.00 |  |  | 0 | 0.00 |  |  | 0 | 0.00 |  |  |
|  |  | AAB | 5 | 9.09 |  |  | 4 | 11.11 |  |  | 5 | 7.69 |  |  |
|  |  | ABB | 5 | 9.09 |  |  | 2 | 5.56 |  |  | 3 | 4.62 |  |  |
|  |  | BBB | 0 | 0.00 |  |  | 0 | 0.00 |  |  | 0 | 0.00 |  |  |
| *FCGR2B* | rs1050501 | II | 46 | 83.64 | 0.37 | 0.79 | 32 | 88.89 | 0.56 | 0.35 | 56 | 86.15 | 0.46 | 0.55 |
|  |  | IT | 9 | 16.36 |  |  | 4 | 11.11 |  |  | 9 | 13.85 |  |  |
|  |  | TT | 0 | 0.00 |  |  | 0 | 0.00 |  |  | 0 | 0.00 |  |  |

HWE was calculated on diploid genotypes within a population. A Chi^2^ <3.5 and a p-value >0.05 were considered within H

**Supplementary Table 5: CNV frequency of Southampton donors with available functional data**

|  | | | **Proliferation cohort** | | **Cytokine cohort** | | **Phenotype cohort** | |
| --- | --- | --- | --- | --- | --- | --- | --- | --- |
| **Gene** | **Copy number** | **N** | | **%** | **N** | **%** | **N** | **%** |
| *FCGR3A* | 1 | 1 | | 1.82 | 1 | 2.78 | 1 | 1.54 |
|  | 2 | 50 | | 90.91 | 33 | 91.67 | 60 | 92.31 |
|  | 3 | 4 | | 7.27 | 2 | 5.56 | 4 | 6.15 |
| *FCGR2C* | 1 | 8 | | 14.55 | 6 | 16.67 | 9 | 13.85 |
|  | 2 | 34 | | 61.82 | 22 | 61.11 | 46 | 70.77 |
|  | 3 | 12 | | 21.82 | 7 | 19.44 | 9 | 13.85 |
|  | 4 | 1 | | 1.82 | 1 | 2.78 | 1 | 1.54 |
| *FCGR3B* | 1 | 7 | | 12.73 | 5 | 13.89 | 8 | 12.31 |
|  | 2 | 38 | | 69.09 | 25 | 69.44 | 49 | 75.38 |
|  | 3 | 10 | | 18.18 | 6 | 16.67 | 8 | 12.31 |
