# Supplementary material for: Impact of Human FcγR Gene Polymorphisms on IgG-Triggered Cytokine Release: Critical Importance of Cell Assay Format
Source: Front Immunol. 2019 Mar 7;10:390. doi: 10.3389/fimmu.2019.00390 (PMC6417454; doi:10.3389/fimmu.2019.00390)
Supplement: Supplementary file 2 [file Presentation_1.PPTX]

## Slide 1
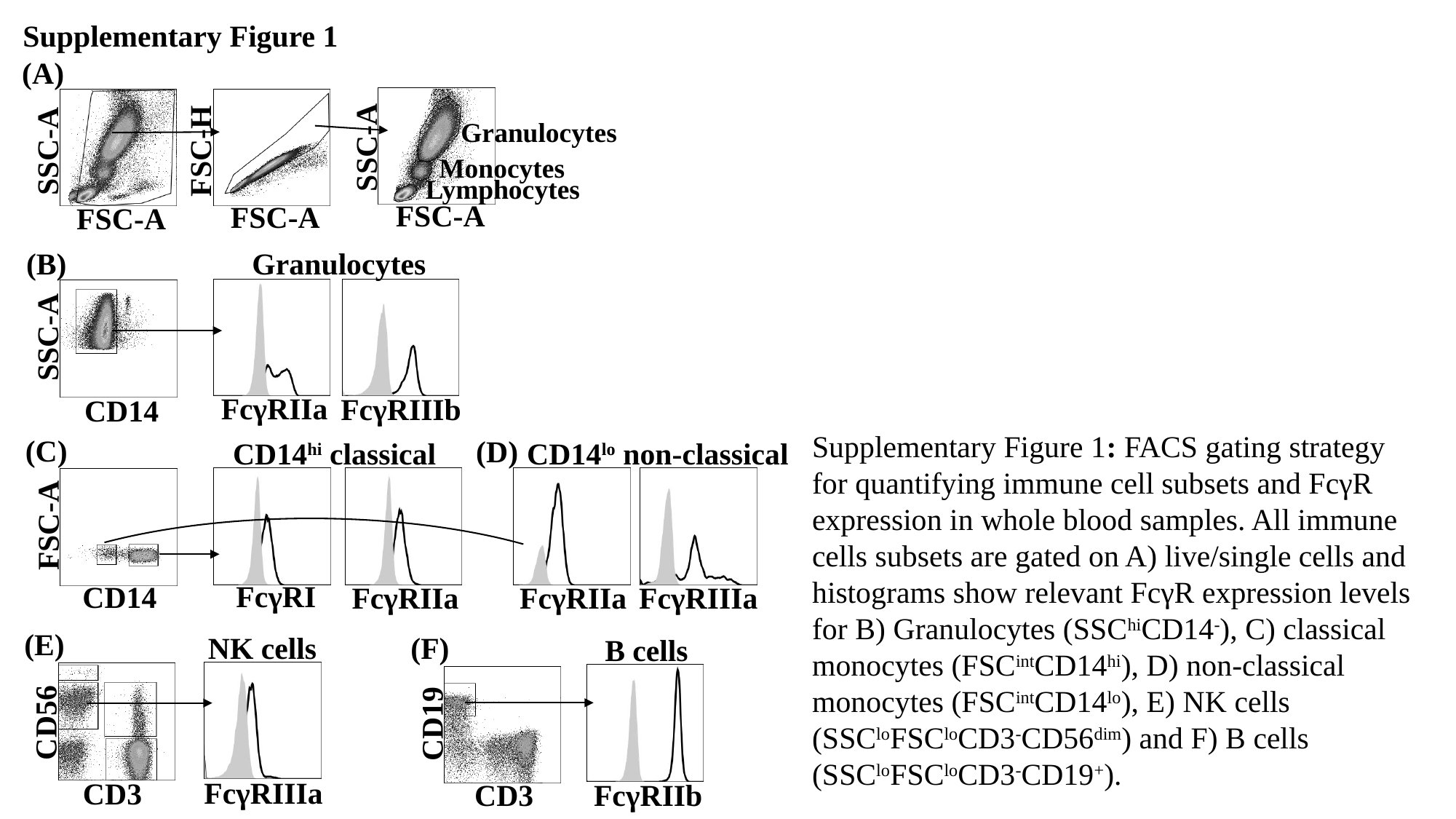

Supplementary Figure 1
(A)
Granulocytes
SSC-A
FSC-H
SSC-A
Monocytes
Lymphocytes
FSC-A
FSC-A
FSC-A
Granulocytes
(B)
SSC-A
FcγRIIa
FcγRIIIb
CD14
Supplementary Figure 1: FACS gating strategy for quantifying immune cell subsets and FcγR expression in whole blood samples. All immune cells subsets are gated on A) live/single cells and histograms show relevant FcγR expression levels for B) Granulocytes (SSChiCD14-), C) classical monocytes (FSCintCD14hi), D) non-classical monocytes (FSCintCD14lo), E) NK cells (SSCloFSCloCD3-CD56dim) and F) B cells (SSCloFSCloCD3-CD19+).
(C)
(D)
CD14lo non-classical
CD14hi classical
FSC-A
FcγRI
CD14
FcγRIIa
FcγRIIa
FcγRIIIa
(E)
(F)
NK cells
B cells
CD56
CD19
FcγRIIIa
CD3
FcγRIIb
CD3

## Slide 2
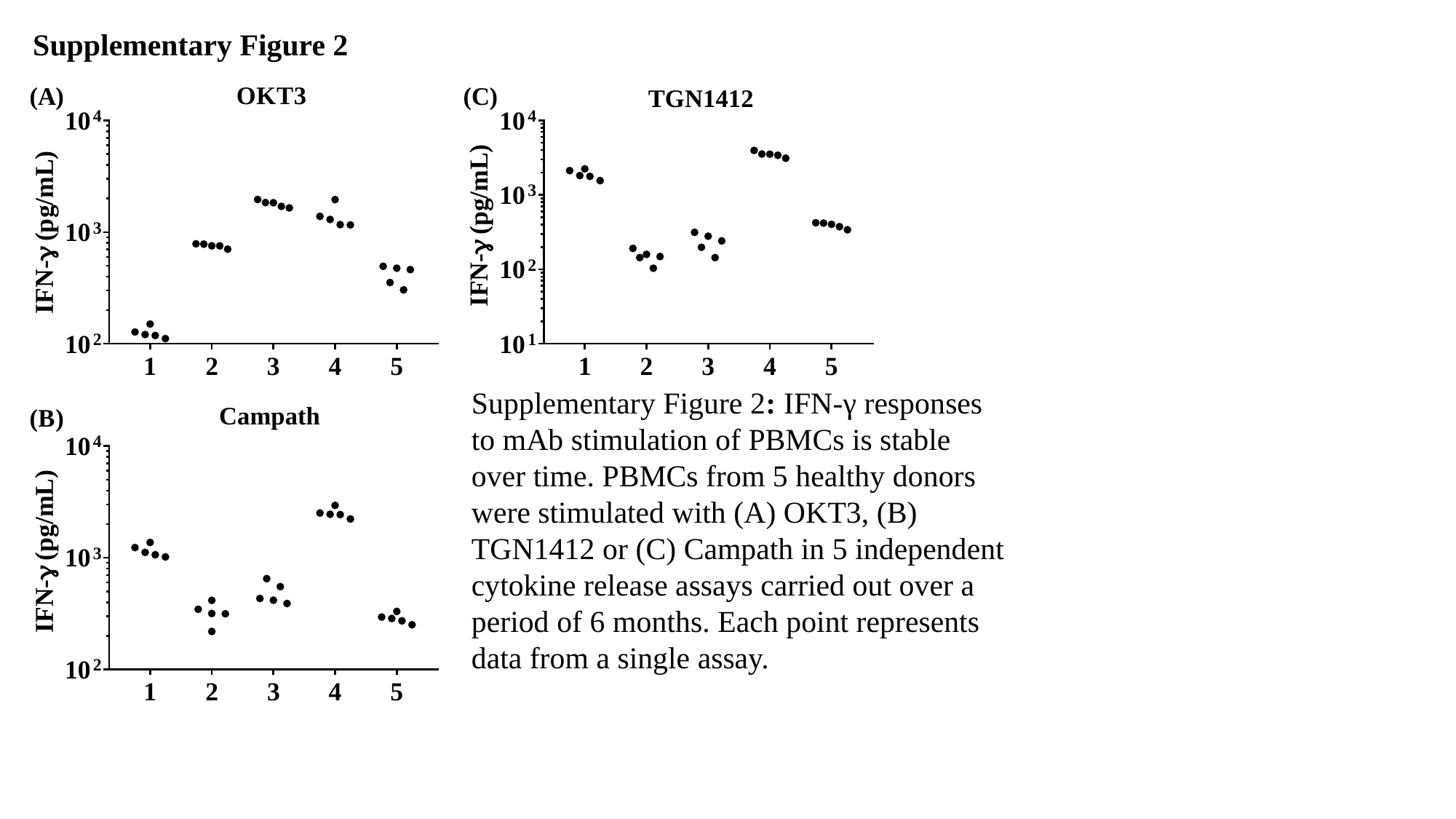

Supplementary Figure 2
Supplementary Figure 2: IFN-γ responses to mAb stimulation of PBMCs is stable over time. PBMCs from 5 healthy donors were stimulated with (A) OKT3, (B) TGN1412 or (C) Campath in 5 independent cytokine release assays carried out over a period of 6 months. Each point represents data from a single assay.

## Slide 3
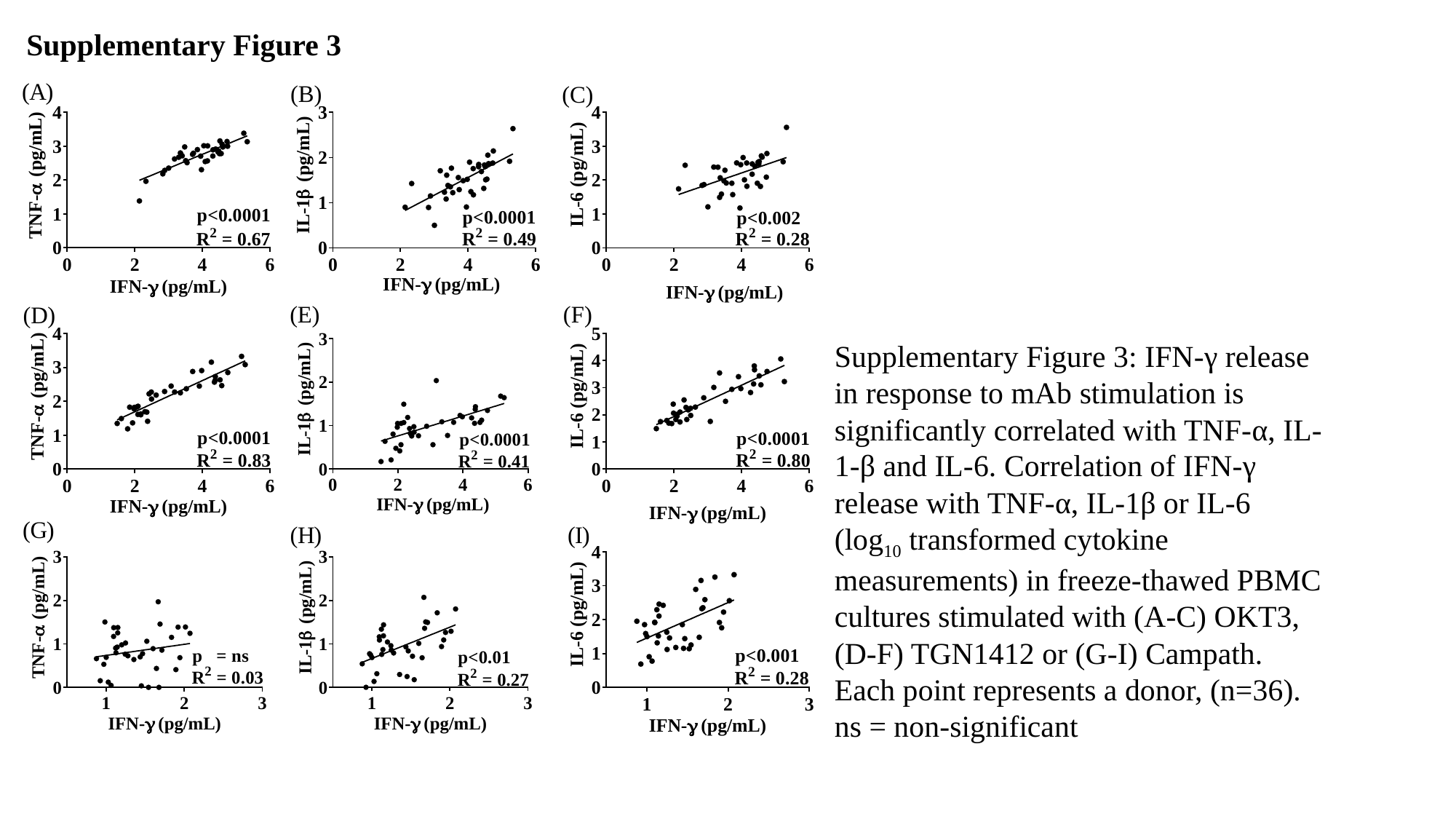

Supplementary Figure 3
Supplementary Figure 3: IFN-γ release in response to mAb stimulation is significantly correlated with TNF-α, IL-1-β and IL-6. Correlation of IFN-γ release with TNF-α, IL-1β or IL-6 (log10 transformed cytokine measurements) in freeze-thawed PBMC cultures stimulated with (A-C) OKT3, (D-F) TGN1412 or (G-I) Campath. Each point represents a donor, (n=36). ns = non-significant

## Slide 4
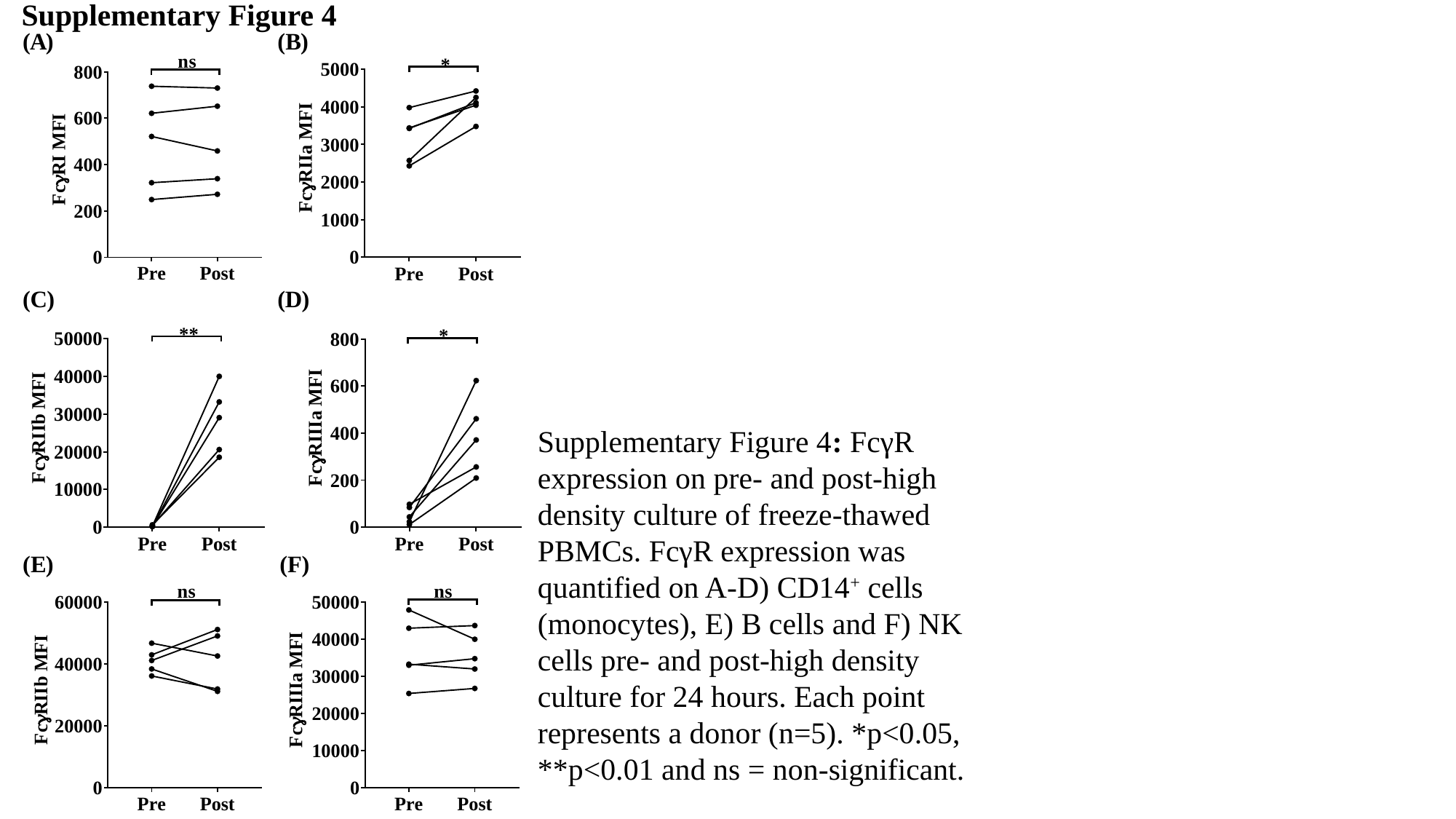

Supplementary Figure 4
Supplementary Figure 4: FcγR expression on pre- and post-high density culture of freeze-thawed PBMCs. FcγR expression was quantified on A-D) CD14+ cells (monocytes), E) B cells and F) NK cells pre- and post-high density culture for 24 hours. Each point represents a donor (n=5). *p<0.05, **p<0.01 and ns = non-significant.

## Slide 5
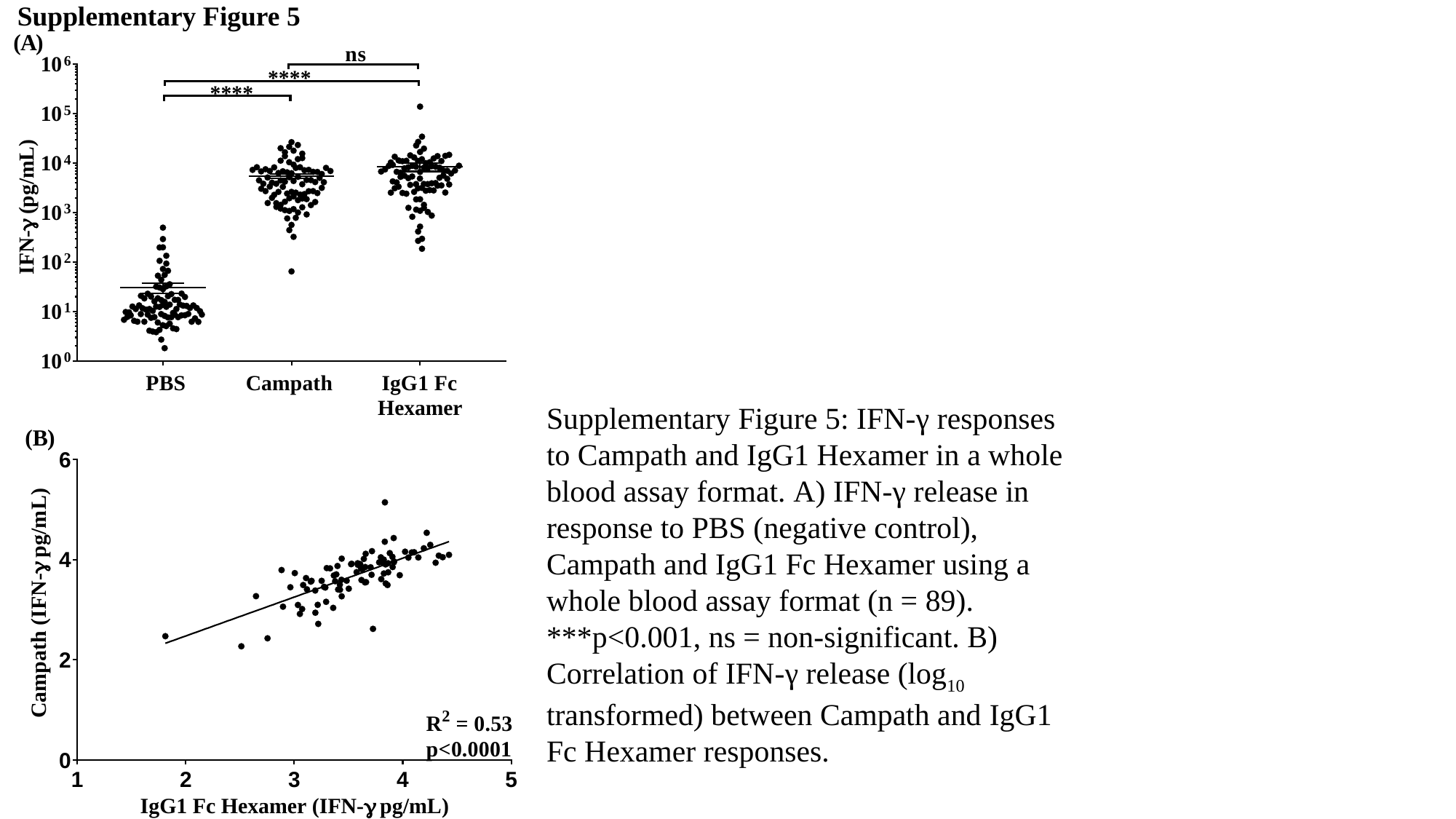

Supplementary Figure 5
Supplementary Figure 5: IFN-γ responses to Campath and IgG1 Hexamer in a whole blood assay format. A) IFN-γ release in response to PBS (negative control), Campath and IgG1 Fc Hexamer using a whole blood assay format (n = 89). ***p<0.001, ns = non-significant. B) Correlation of IFN-γ release (log10 transformed) between Campath and IgG1 Fc Hexamer responses.

## Slide 6
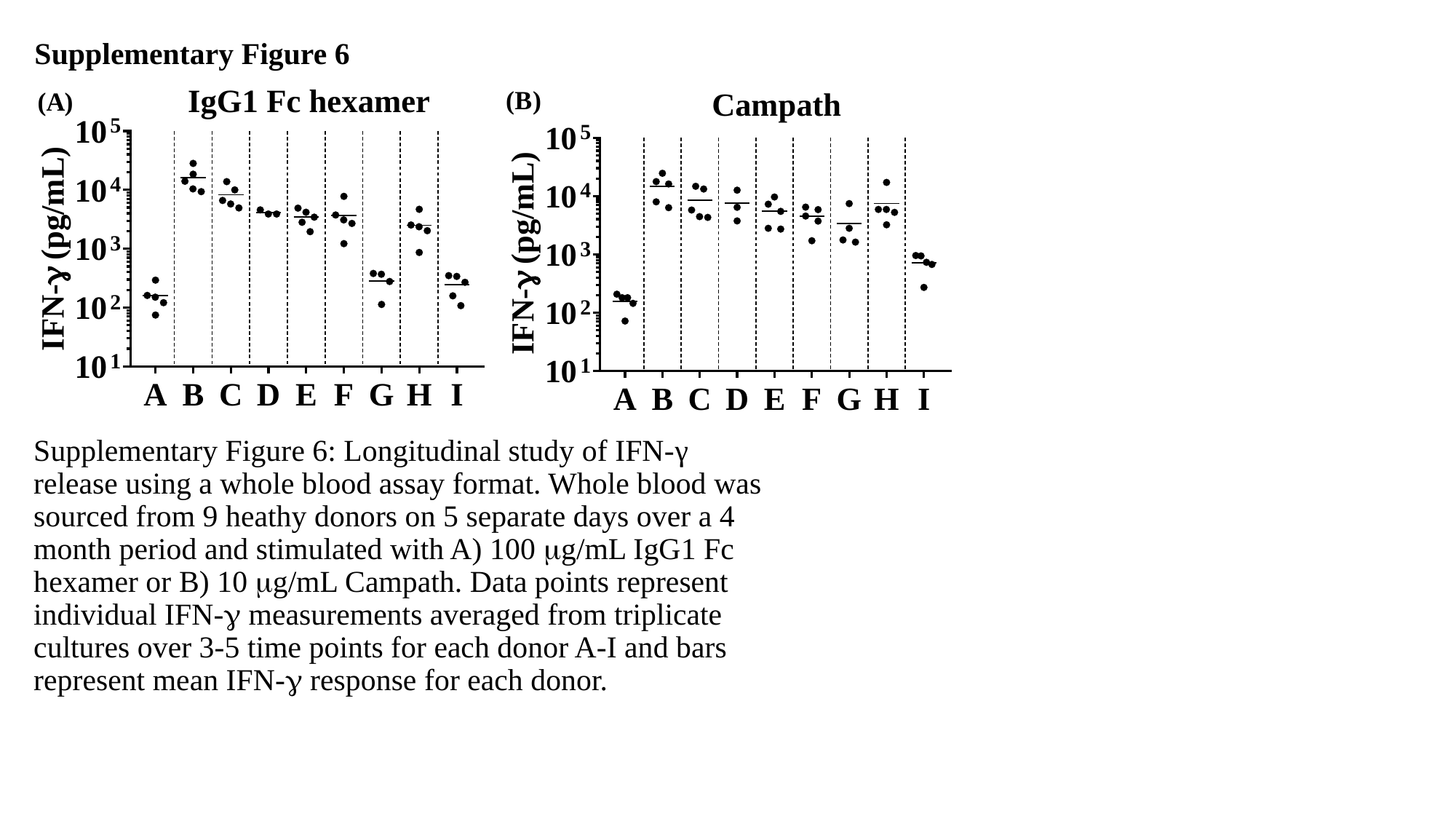

Supplementary Figure 6
# Supplementary Figure 6: Longitudinal study of IFN-γ release using a whole blood assay format. Whole blood was sourced from 9 heathy donors on 5 separate days over a 4 month period and stimulated with A) 100 μg/mL IgG1 Fc hexamer or B) 10 μg/mL Campath. Data points represent individual IFN-γ measurements averaged from triplicate cultures over 3-5 time points for each donor A-I and bars represent mean IFN-γ response for each donor.

## Slide 7
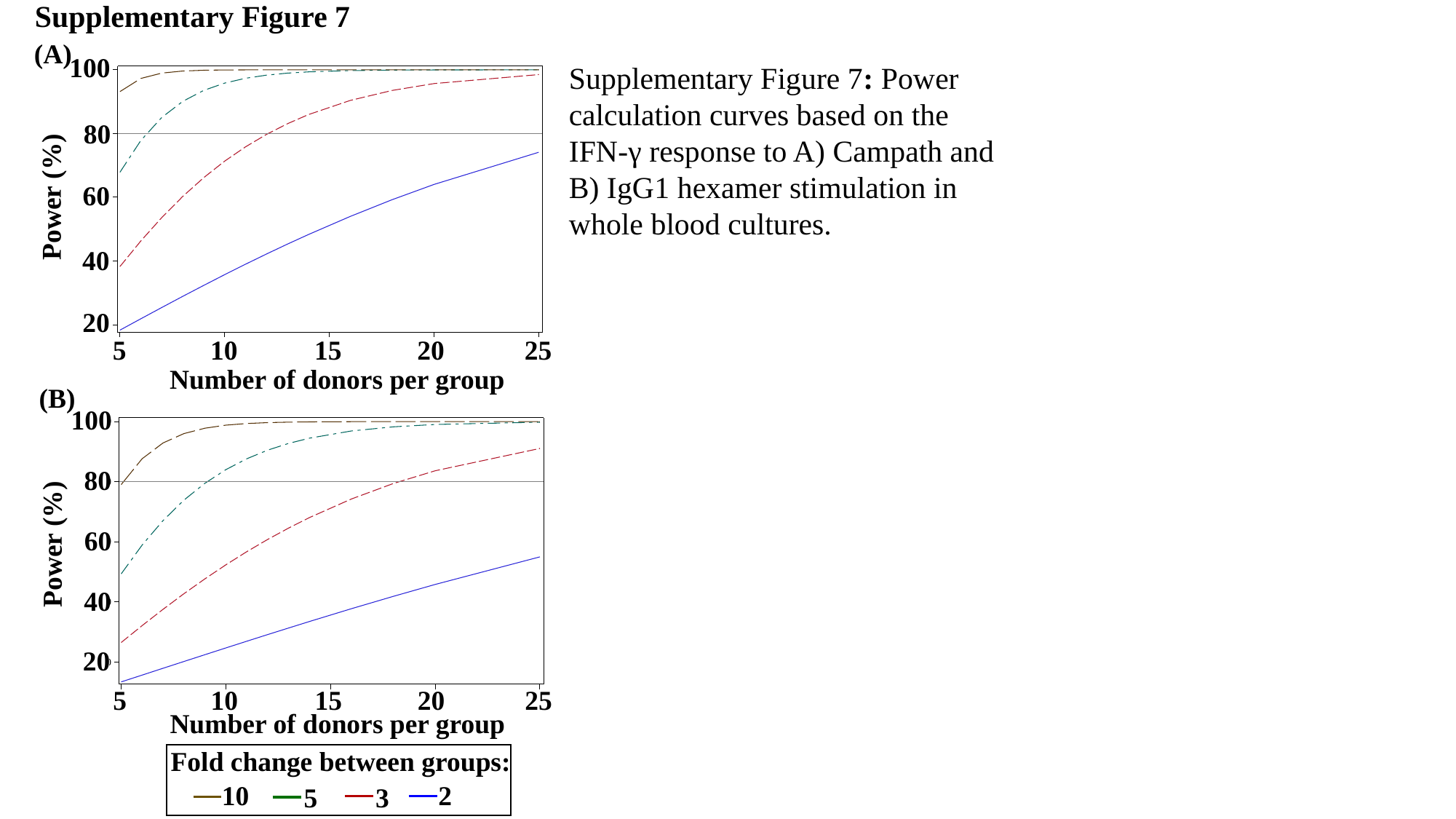

Supplementary Figure 7
(A)
100
80
60
Power (%)
40
20
5
10
15
20
25
Number of donors per group
(B)
100
80
60
Power (%)
40
20
5
10
15
20
25
Number of donors per group
Fold change between groups:
10
2
5
3
Supplementary Figure 7: Power calculation curves based on the IFN-γ response to A) Campath and B) IgG1 hexamer stimulation in whole blood cultures.
